# Supplementary material for: Tollip coordinates Parkin‐dependent trafficking of mitochondrial‐derived vesicles
Source: EMBO J. 2020 Apr 20;39(11):e102539. doi: 10.15252/embj.2019102539 (PMC7265236; doi:10.15252/embj.2019102539)
Supplement: Supplementary file 1 — Appendix [file EMBJ-39-e102539-s001.pdf]

Appendix

Tollip coordinates Parkin-dependent trafficking of mitochondrial-derived vesicles

Thomas A. Ryan, Elliott O. Phillips, Charlotte L. Collier, Alice J. B. Robinson, Daniel Routledge, Rebecca E. Wood, Emelia A. Assar, David A. Tumbarello

Table of Contents

Appendix Figure S1: Tollip depletion does not alter mitochondrial localisation of Parkin or PINK1.

Appendix Figure S2: Tollip and Parkin colocalise on a vesicular compartment during mitochondrial stress.

Appendix Figure S3:TOM20 MDVs form independent of Parkin E3 ligase activity.

Appendix Figure S4: Tollip is trafficked through Rab5 and Rab7 compartments during mitochondrial stress, but does not localise to the mitochondrial network or Golgi apparatus.

Appendix Figure S5: Lysosomal trafficking of Tollip is dependent on Tom1, but independent of autophagosomes.

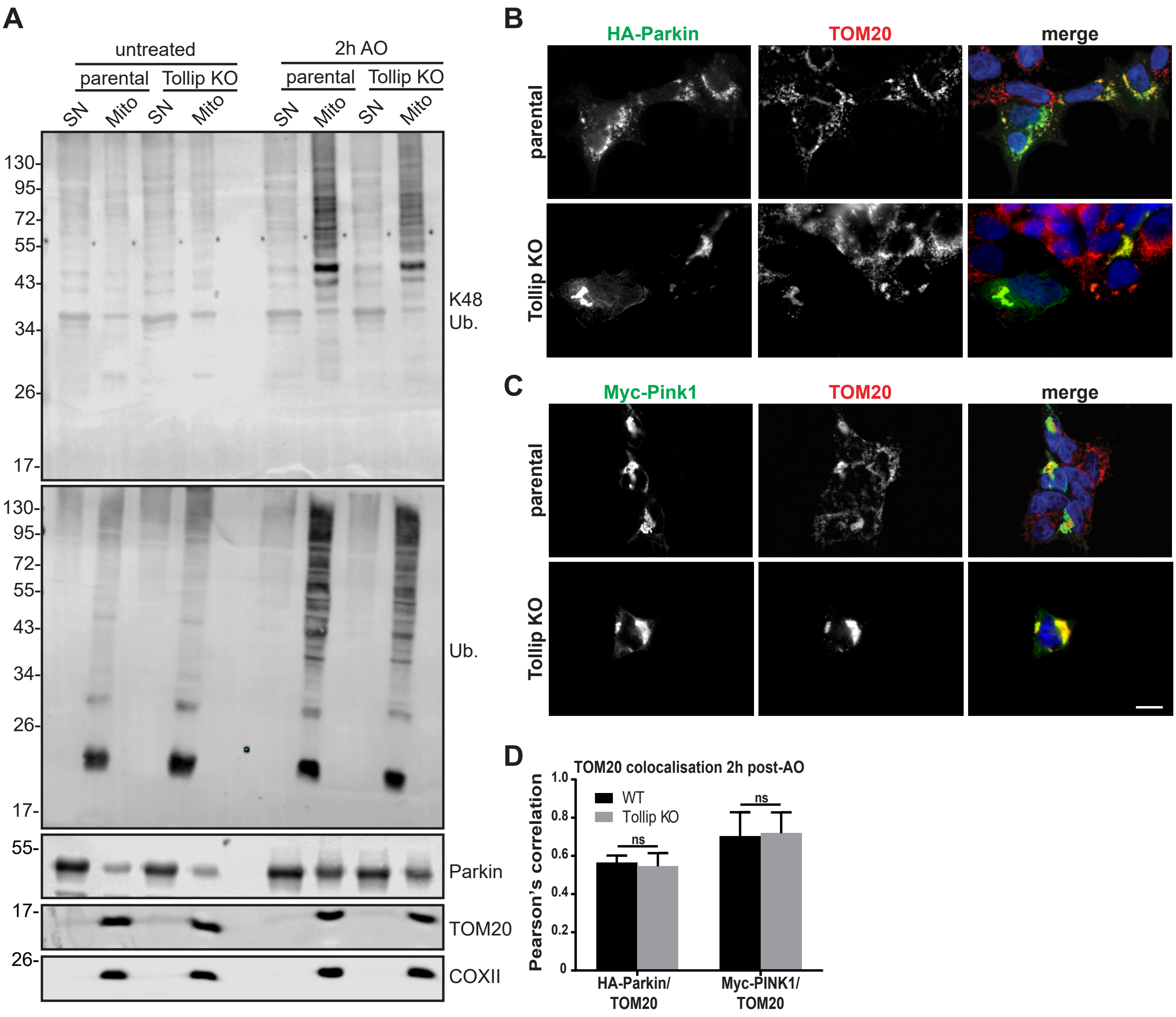

Figure S1: Tollip depletion does not alter mitochondrial localisation of Parkin or PINK1. (A) HEK293 cells (parental and Tollip KO) expressing HA-Parkin were treated with AO for 2 hours, then cells were lysed. Mitochondrial fractions (mito) were isolated and analysed by western blot analysis alongside supernatants (SN) obtained after mitochondrial extractions. Knockout of Tollip did not appear to alter Parkin translocation to mitochondria following AO, or subsequent ubiquitylation. (B-D) HEK293 cells (parental and Tollip KO) expressing HA-Parkin or Myc-PINK1 were analysed by immunofluorescence microscopy 2 hours after AO treatment. Knockout of Tollip did not alter the translocation of exogenous Parkin or PINK1 to mitochondria (TOM20), which was assessed by colocalisation analysis represented by a Pearson's correlation (2-3 cells/experiment, from 3 independent experiments). Statistical significance was assessed using a 2-way ANOVA followed by Dunnett's multiple comparison test (ns = not significant). Bars indicate the mean and error bars represent SEM. Scale bar represents 10 μm.

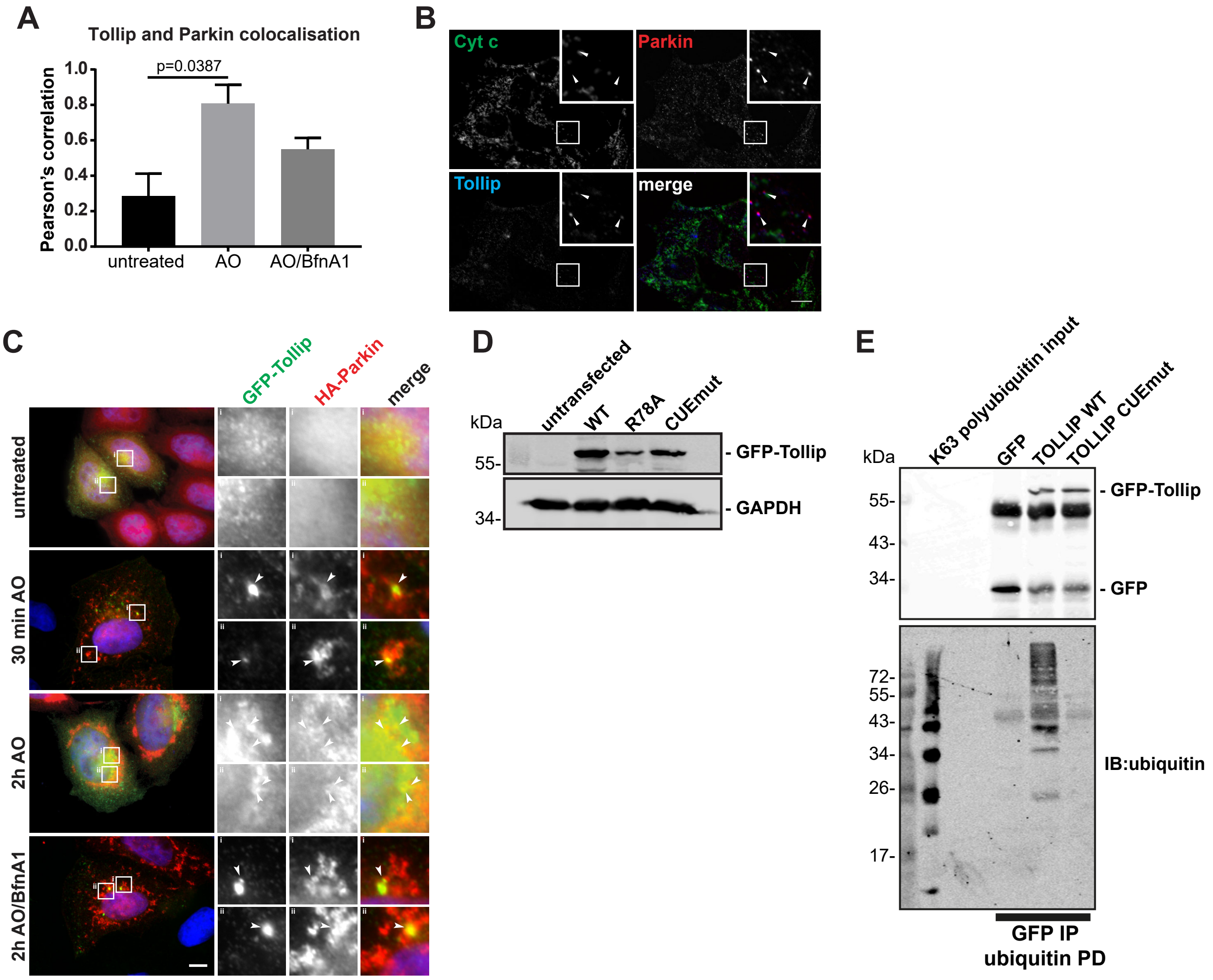

Figure S2: Tollip and Parkin colocalise on a vesicular compartment during mitochondrial stress. (A) Quantification of GFP-Tollip/Parkin colocalisation by Pearson's correlation (2-3 cells/experiment, from 3 independent experiments). Statistical significance was determined using a One way ANOVA (Tukey). Bars indicate the mean and error bars represent SEM. (B) A subset of endogenous Tollip and Parkin colocalised in SH-SY5Y cells. Cells were treated with 5 µM antimycin A/10 µM oligomycin B (AO) for 2 hours, then fixed and immunostained with antibodies specific to cytochrome c (green), Parkin (red) and Tollip (blue). Zoom insets represent 3.5x magnification and arrowheads indicate Tollip and Parkin colocalisation. (C) HeLa cells transfected with GFP-Tollip (WT) and HA-Parkin were treated with AO or AO/100 nM bafilomycin A (AO/BfnA1) prior to fixation and immunostaining with antibodies specific to GFP (green) and Parkin (red). Arrowheads illustrate Parkin and Tollip colocalisation. Images were captured by widefield immunofluorescence microscopy and zoom insets represent 5x magnification. All scale bars represent 10 µm. (D) SH-SY5Y cells were transiently transfected with GFP-Tollip wild-type (WT), R78A, and CUEmut followed by western blot analysis. Immunoblotting was performed using antibodies against GFP and GAPDH. (E) GFP alone, GFP-Tollip WT and GFP-Tollip CUEmut were immunoprecipitated from Hela cells prior to incubation with K63 polyubiquitin for pulldown assay. Western blot analysis was performed on GFP pulldowns alongside K63 polyubiquitin input and probed with antibodies against GFP and ubiquitin. GFP-Tollip WT interacted with polyubiquitin, whereas the Tollip CUE mutant did not.

Ryan et al., Figure S2

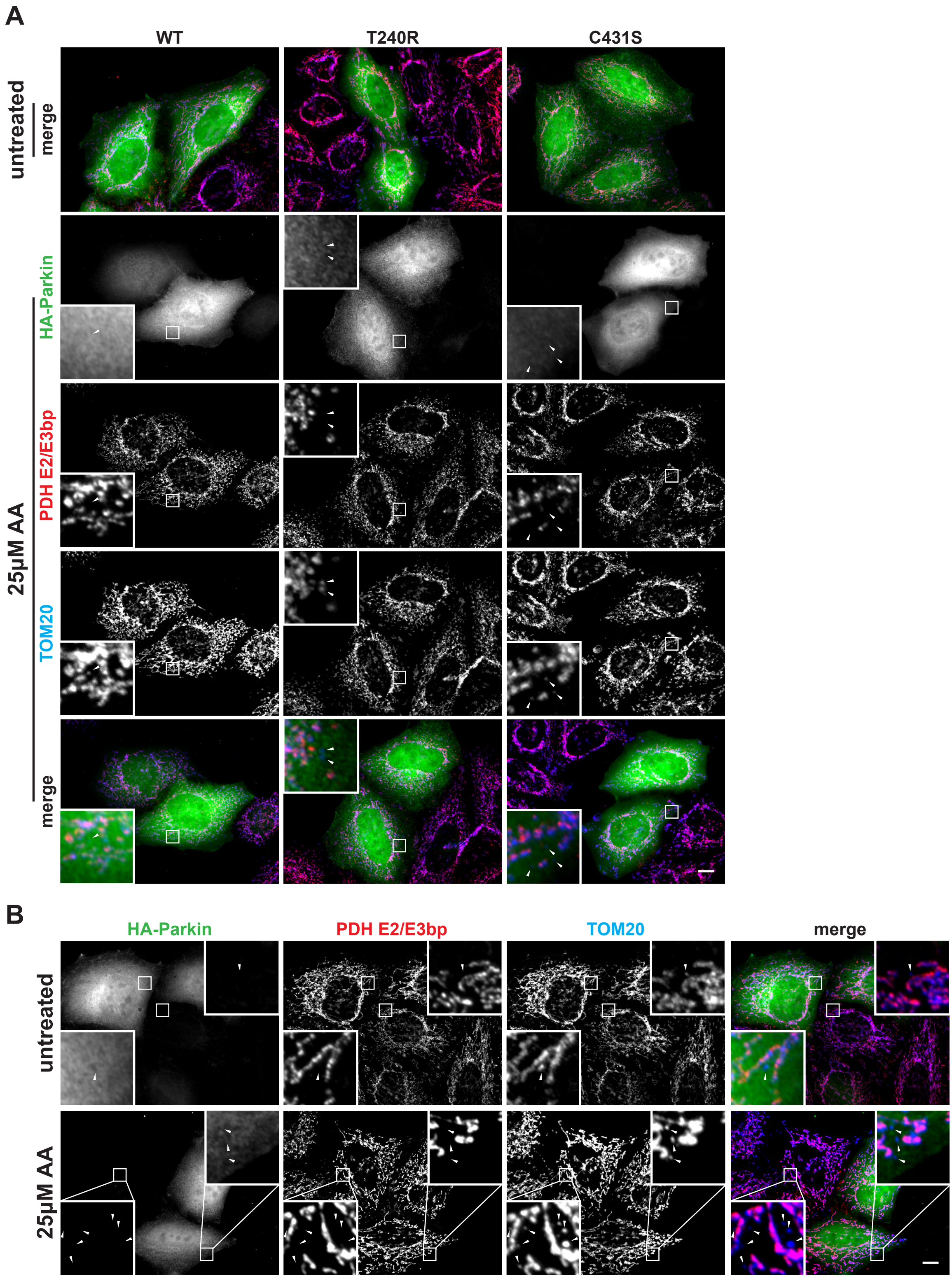

Figure S3: TOM20 MDVs form independent of Parkin E3 ligase activity. HeLa cells expressing HA-tagged Parkin WT or E3-deficient mutants (T240R or C431S) were subjected to mitochondrial stress induction by 25 µM antimycin A (AA) for 2 hours. Cells were then fixed and immunostained with antibodies specific to HA (green), PDH (red) and TOM20 (blue). TOM20+ve/PDH-ve MDVs are denoted by arrowheads. (A) These MDVs were observed in cells expressing T240R or C431S Parkin mutants, indicating that MDV formation does not require Parkin E3 ligase activity. (B) Expression of HA-Parkin WT reduced the number of TOM20 MDVs observed in cells undergoing mitochondrial stress (arrowheads). Scale bars represent 10 µm. Zoom insets represent 6.5x magnification.

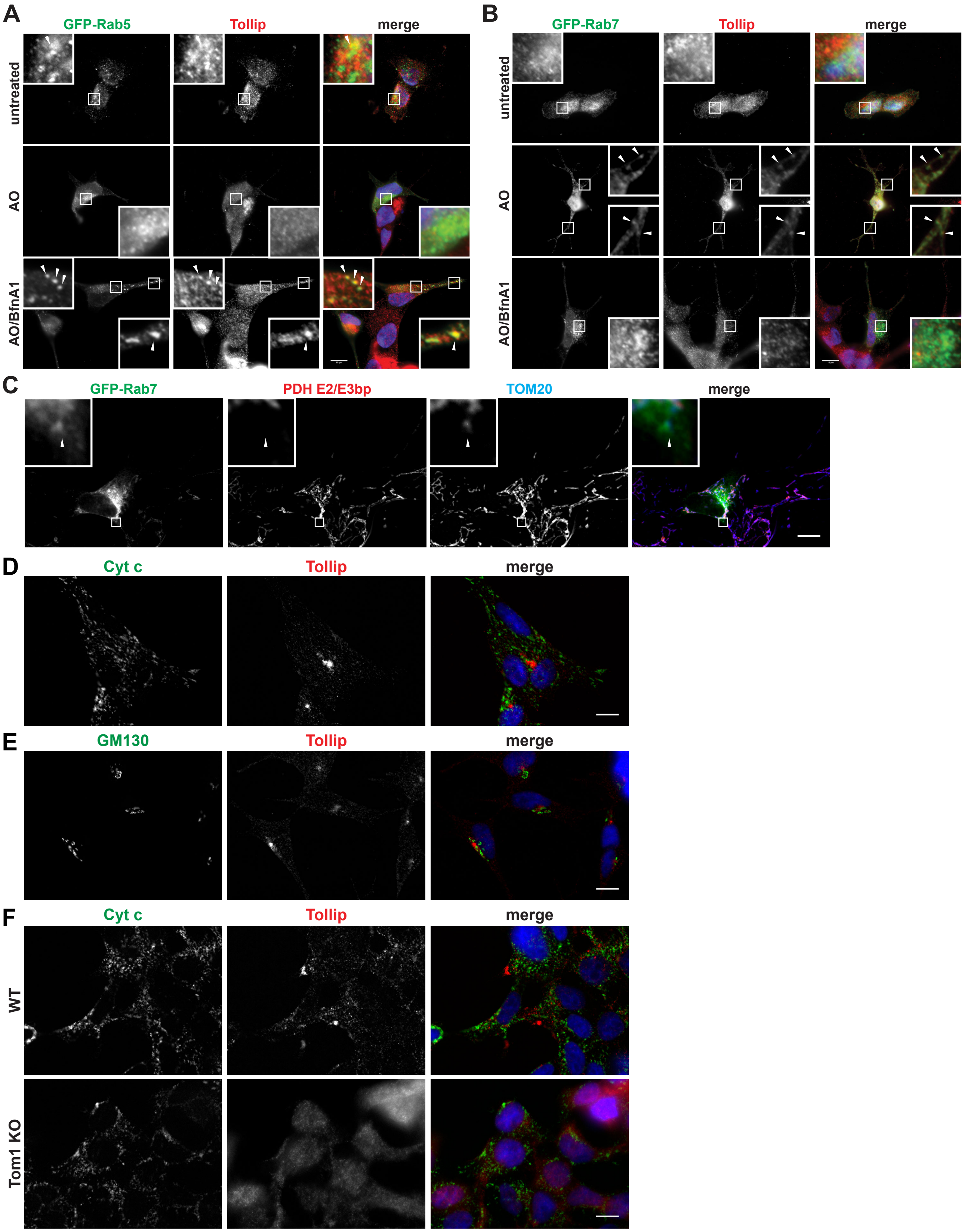

Figure S4: Tollip is trafficked through Rab5 and Rab7 endosomal compartments during mitochondrial stress, but does not localise to the mitochondrial network or Golgi apparatus. SH-SY5Y cells were transfected with GFP-Rab5 (A) or GFP-Rab7 (B) prior to the induction of mitochondrial stress by 5 μM antimycin A/10 μM oligomycin B (AO) or AO/100 nM Bafilomycin A1 (AO/BfnA1) for 2 hours. Cells were then fixed and immunostained with antibodies specific to GFP (green) and Tollip (red). (A) A subset of Tollip localised to Rab5-positive vesicles in untreated cells. This colocalisation (denoted by arrow-heads) was not observed during AO-induced stress, but accumulated in cells undergoing both mitochondrial stress and vATPase inhibition. (B) Tollip was observed in Rab7-positive vesicles during mitochondrial stress (arrowheads). Zoom insets in (A) and (B) represent 5x magnification. (C) A subset of TOM20 MDVs were positive for GFP-Rab7 (arrowhead). SH-SY5Y cells were transiently transfected and fixed prior to immunostaining with antibodies specific to GFP (green), PDH (red) and TOM20 (blue). Zoom insets represent 9x magnification. (D-E) SH-SY5Y cells were treated with 5 μM antimycin A/10 μM oligomycin B (AO) for 2 hours to induce Tollip perinuclear clustering. Cells were then fixed and immunostained with antibodies specific to Cytochrome c (mitochondria, green) and Tollip (red) or GM130 (Golgi, green) and Tollip (red). Tollip clustering did not colocalise with either the mitochondria or Golgi. (F) HEK293 WT or Tom1 KO cells were treated with AO for 2 hours to induce Tollip perinuclear clustering. Cells were immunostained with antibodies specific to Cytochrome c (green) and Tollip (red). Clustering observed in WT cells was absent in Tom1 KO cells. Images were captured at 100X magnification. Scale bars represent 10 μm.

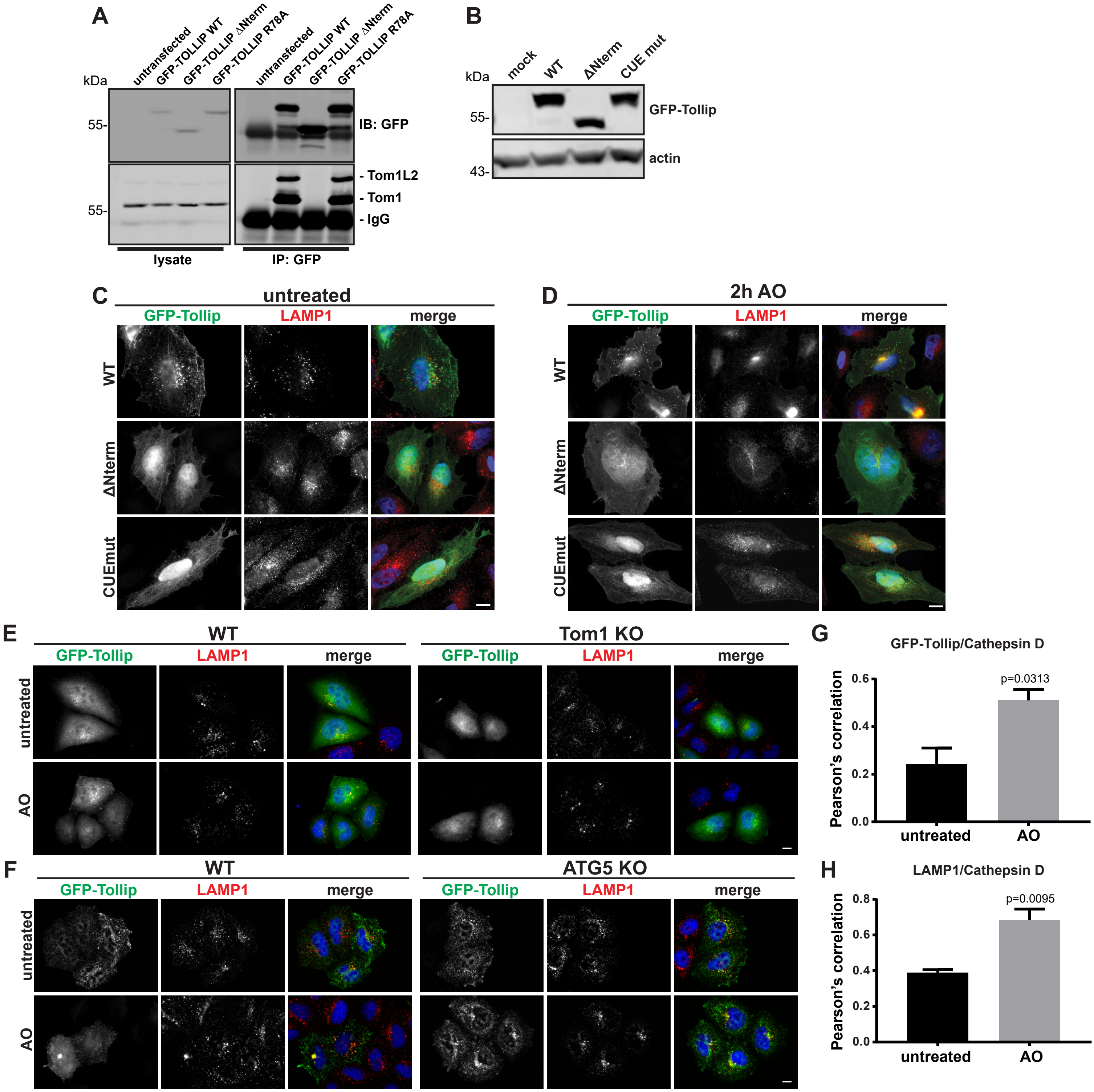

Figure S5: Lysosomal trafficking of Tollip is dependent on Tom1, but independent of autophagosomes. (A) RPE-1 cells were transfected with GFP-labelled Tollip WT, ΔNterm or R78A. GFP-Tollip WT and R78A co-immunoprecipitated with Tom1, as well as Tom1L2, whereas the ΔNterm did not. (B) HeLa cells were transfected with GFP-labelled Tollip WT, ΔNterm and CUE mutant for 24 hours then lysed and subjected to western blotting. Expression of all three constructs was observed at similar levels and, as expected, the N-terminal deletion demonstrated a lower molecular weight. (C-D) HeLa cells were transfected with GFP-Tollip WT, ΔNterm or CUE mutant constructs 24 hours prior to treatment with 5 μM antimycin A/10 μM oligomycin B (AO) for 2 hours. Cells were then fixed and immunostained with antibodies specific to GFP (green) and LAMP1 (red). (E) HeLa WT and Tom1 KO cells were transfected with GFP-Tollip 24 hours prior to the induction of mitochondrial stress by 5 μM antimycin A/10 μM oligomycin B (AO) for 2 hours. Cells were then fixed and immunostained with antibodies specific to GFP (green) and LAMP1 (red). Translocation of GFP-Tollip observed in WT cells during mitochondrial stress was abrogated by Tom1 KO. (F) HeLa WT and ATG5 KO cells were transfected with GFP-Tollip 24 hours prior to the induction of mitochondrial stress AO for 2 hours. Cells were then fixed and immunostained with antibodies specific to GFP (green) and LAMP1 (red). GFP-Tollip trafficking to LAMP1 vesicles was maintained in ATG5 KO cells, indicating that it is not delivered via an autophagosomal compartment. Images were captured at 63X magnification. Scale bars represent 10 μm. (G-H) SH-SY5Y cells were transfected with GFP-Tollip 24 hours prior to treatment with AO for 2 hours. Cells were then fixed and immunostained with antibodies specific to LAMP1 and Cathepsin D. Immunofluorescence images were then captured at 63X magnification. Colocalisation of GFP-Tollip and Cathepsin D (G) or LAMP1 and Cathepsin D (H) was assessed by Pearson's correlation (2-3 cells/experiment, from 3 independent experiments). Statistical significance was determined using an unpaired t-test, bars indicate the mean, and error bars represent the SEM.
