# Supplementary material for: Tollip coordinates Parkin‐dependent trafficking of mitochondrial‐derived vesicles
Source: EMBO J. 2020 Apr 20;39(11):e102539. doi: 10.15252/embj.2019102539 (PMC7265236; doi:10.15252/embj.2019102539)
Supplement: Supplementary file 2 — Expanded View Figures PDF [file EMBJ-39-e102539-s002.pdf]

Expanded View Figures

Figure EV1. Parkin and Tollip are both associated with mitochondrial quality control.

A SH-SY5Y cells were seeded on glass coverslips for 24 h and then treated with 5 μM antimycin/10 μM oligomycin for 2 or 6 h prior to fixation. Cells were immunostained with antibodies specific to LAMP1 (green) to define late endosomes/lysosomes, Parkin (red) and TOM20 (blue) to define mitochondria. Images were captured by widefield immunofluorescence microscopy at 63× magnification. Zoom insets represent 3.5× magnification.

B HEK293 WT and Tollip KO cells were immunostained for PDH (green) and TOM20 (red). Nuclei were labelled with Hoechst (blue). A number of $TOM20^{+ve}/PDH^{-ve}$ MDVs were observed in Tollip KO cells (arrowheads). Images were captured at 100× magnification. Zoom insets represent 5× magnification. Western blot analysis of lysates harvested from HEK293 parental and Tollip KO cells, immunoblotted with antibodies specific to Tollip and actin.

C HeLa cells expressing HA-Parkin were transfected with GFP-Tollip 24 h prior to treatment with 5 μM antimycin/10 μM oligomycin (AO) or 25 μM antimycin A (AA) for 2 h. Cells were fixed and stained for GFP (green), PDH (red) and TOM20 (blue). GFP-Tollip was observed to localise to a small subset of TOM20-positive MDVs (arrowheads). Images were captured at 100× magnification. Zoom insets represent 4× magnification.

Data information: Scale bars represent 10 μm.
Source data are available online for this figure.

© 2020 The Authors

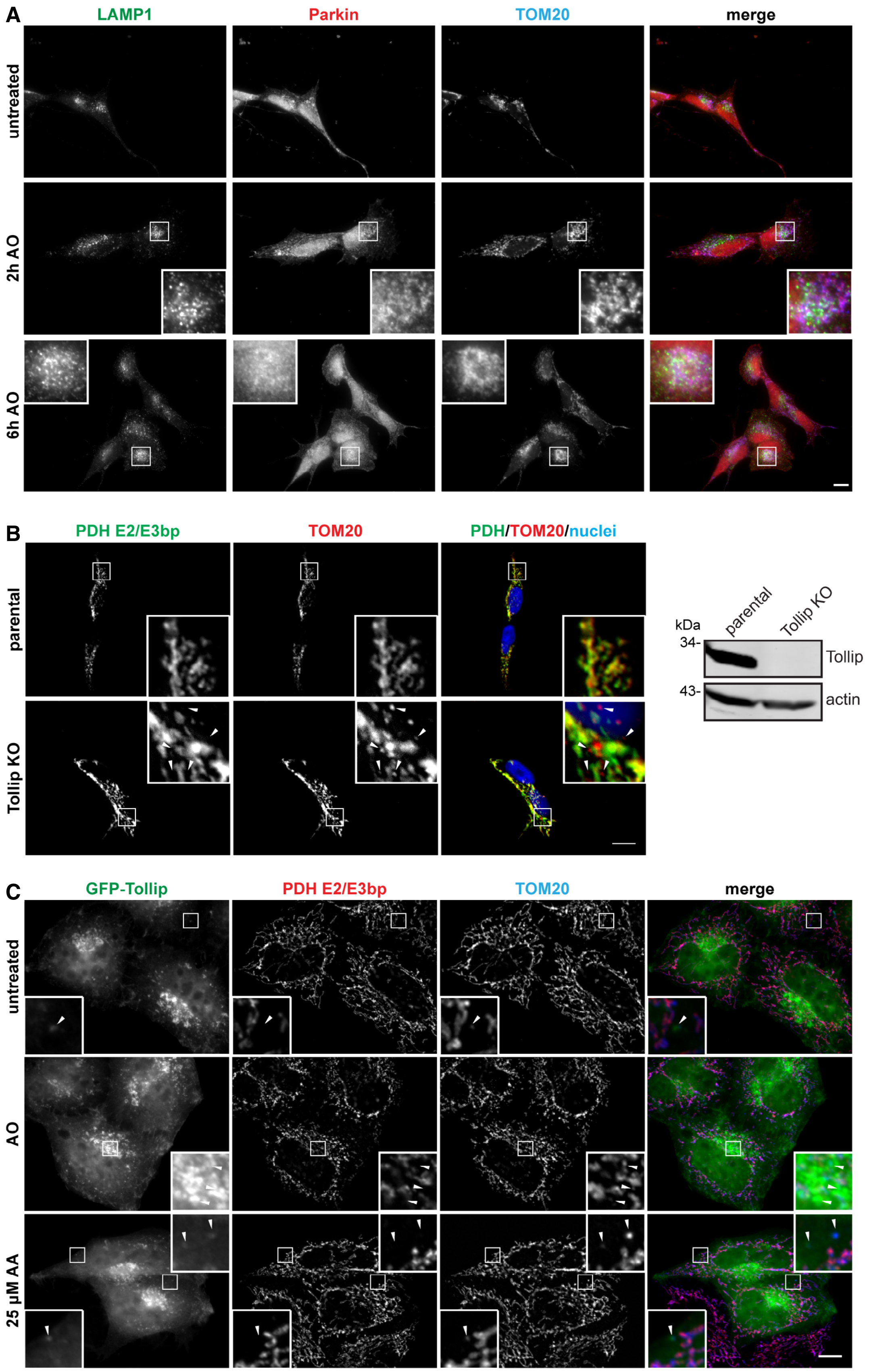
A
LAMP1
Parkin
TOM20
merge
untreated
2h AO
6h AO
B
PDH E2/E3bp
TOM20
PDH/TOM20/nuclei
parental
Tollip KO
kDa
parental
Tollip KO
34-
Tollip
43-
actin
C
GFP-Tollip
PDH E2/E3bp
TOM20
merge
untreated
AO
25 µM AA

Figure EV1.

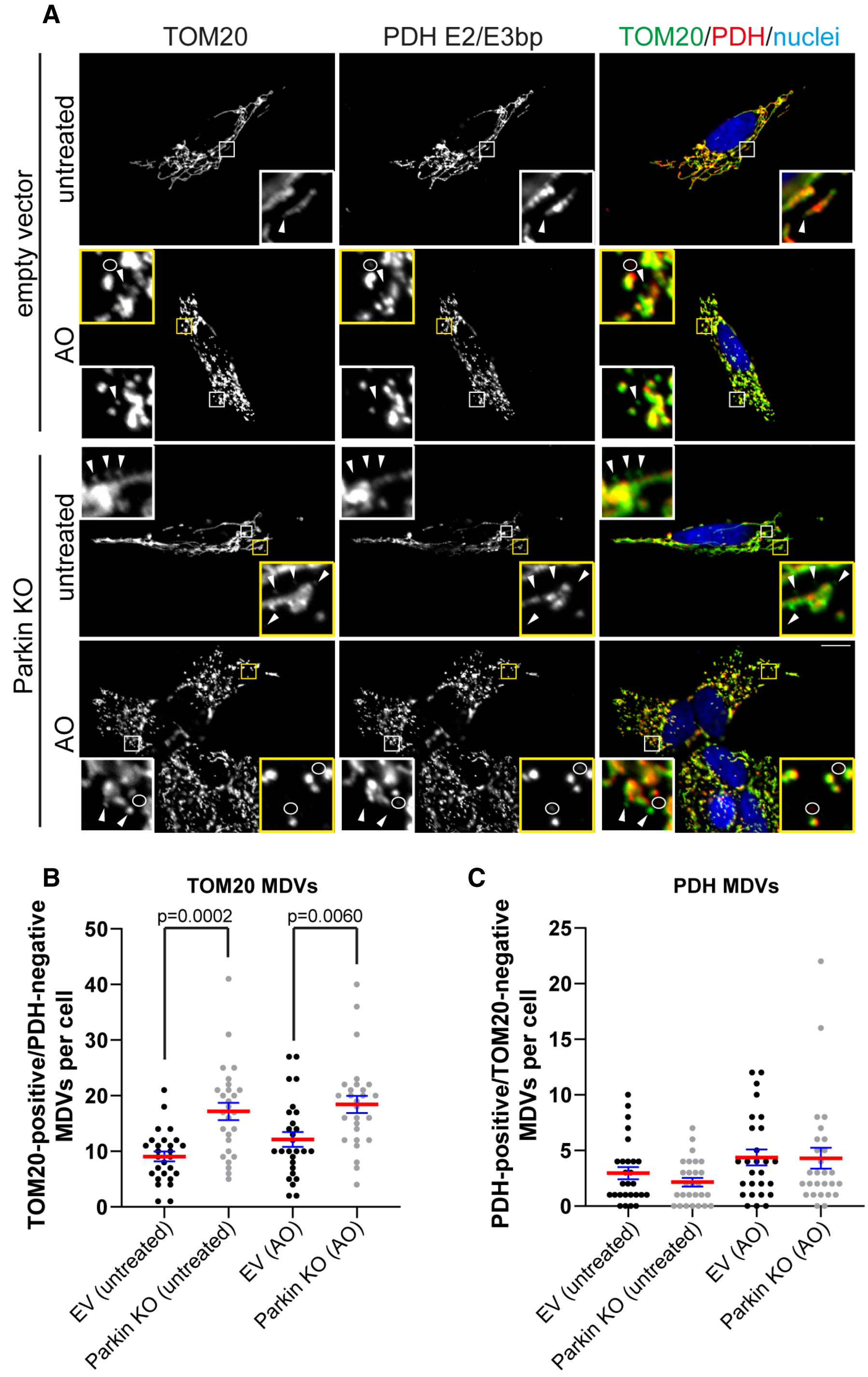

Figure EV2. Knockout of Parkin expression in SH-SY5Y cells results in the selective accumulation of TOM20-positive MDVs.

A SH-SY5Y empty vector (EV) or Parkin KO cells were untreated or treated with AO for 2 h prior to processing for immunofluorescence microscopy. Cells were immunostained for TOM20 (green) and PDH E2/E3 bp (red). Nuclei were labelled with Hoechst (blue). Arrowheads indicate TOM20-positive MDVs, and circles identify PDH-positive MDVs. Scale bar represents 10 μm. Zoom insets represent 5× magnification.

B, C Quantitation of the number of TOM20-positive (B) and PDH-positive (C) MDVs per cell were performed from three independent experiments (27 total cells/condition). Statistical significance was determined using a two-way ANOVA followed by Tukey's multiple comparison test. Centre line indicates the mean, and error bars represent the SEM.

 © 2020 The Authors

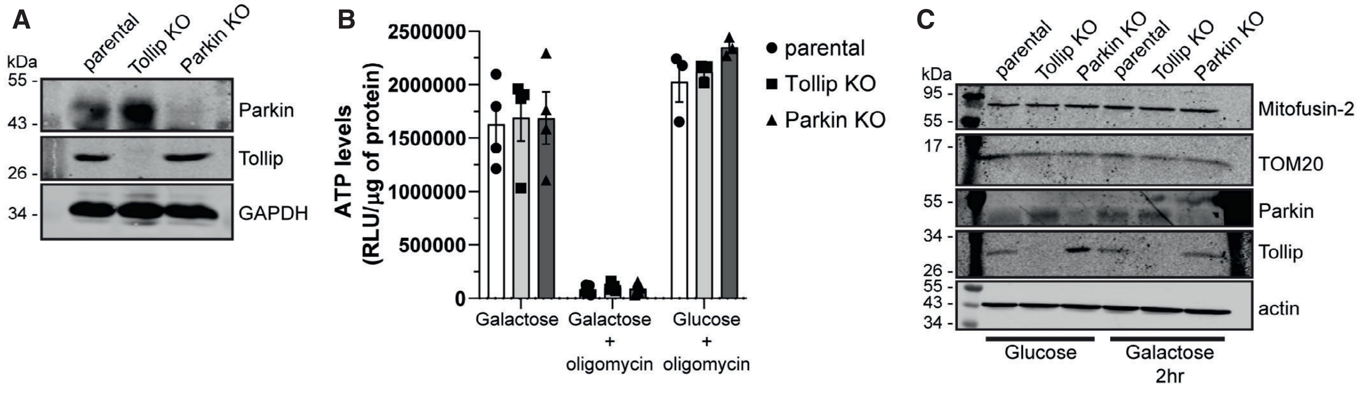

Figure EV3. Loss of Tollip and Parkin expression has no effect on mitochondrial ATP production.

- A Western blot analysis of lysates harvested from SH-SY5Y parental (wild type), Tollip KO and Parkin KO cells was performed using antibodies specific to the indicated proteins.
- B SH-SY5Y parental, Tollip KO and Parkin KO cells were cultured in Galactose media in the absence or presence of 10 µm oligomycin, or in glucose-containing media in the presence of oligomycin. Cells were incubated for 2 h prior to harvesting for either measurement of luminescence signal proportional to ATP present or total protein content. Data represent mean ATP levels as measured by relative light units (RLUs)/µg of protein. Data represent 3–4 independent experiments/condition (indicated by data points), and error bars represent SEM.
- C Western blot analysis of mitochondrial resident protein expression from lysates harvested from SH-SY5Y parental, Tollip KO or Parkin KO cells cultured in glucose or galactose media for 2 h. Immunoblotting was performed against the indicated proteins.

Source data are available online for this figure.

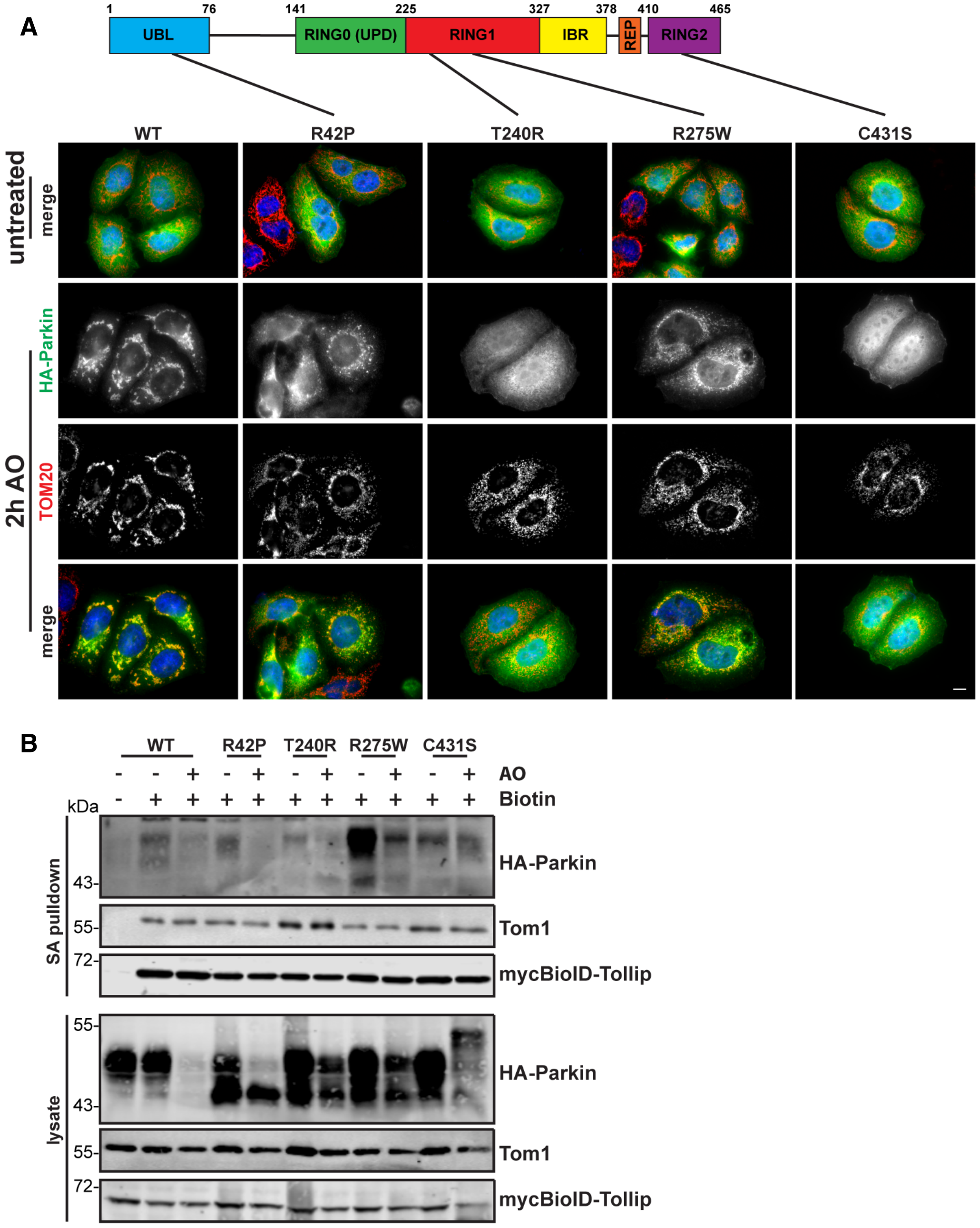

Figure EV4. Parkin mutants that affect ligase activity, autoinhibition and translocation to mitochondria retain an interaction with Tollip.

A Schematic of Parkin detailing the point mutations assessed here. Parkin is divided into the ubiquitin-like (UBL) domain, the RING0 or unique Parkin domain (UPD), a RING1 domain, the in-between ring-finger (IBR) domain, the repressor element of Parkin (REP) and a RING2 domain. HeLa cells expressing wild type and various HA-Parkin mutants were treated for 2 h with 5 μM antimycin A/10 μM oligomycin (AO) for 2 h, then fixed and immunostained for Parkin (green) and TOM20 (red). Under steady-state conditions, all Parkin mutants were cytosolic. However, WT, R42P and R275W constructs were recruited to damaged mitochondria after AO treatment, whereas T240R and C431S remained cytosolic. Scale bar represents 10 μm.

B HeLa cells expressing mycBioID–Tollip and HA-Parkin mutants were treated with AO for 18 h in the presence of biotin. Cells were lysed and streptavidin pulldowns performed overnight to isolate biotinylated proteins. Proteins in whole-cell extracts and pulldowns from each condition were then separated by SDS–PAGE and membranes probed for indicated proteins. Pulldowns of Tom1 and mycBioID–Tollip were used as positive controls. Cells cultured in media lacking biotin were used as a negative control to assess background levels.

Source data are available online for this figure.

 © 2020 The Authors

Figure EV5. VPS35 colocalises with Tollip and maintains lysosomal integrity, while its loss of function has no effect on Tollip–Parkin interactions.

A SH-SY5Y cells were transfected with GFP-Tollip and then treated with 5 μM antimycin A/10 μM oligomycin (AO) or AO/100 μM bafilomycin A (BfnA1) for 2 h prior to fixation and immunostaining. Antibodies specific to GFP (green) and VPS35 (red) were used. Zoom insets represent 4× magnification.

B Quantification of GFP-Tollip/VPS35 colocalisation was assessed by Pearson's correlation from two independent experiments. Each data point represents 1 cell, and bar indicates mean value (7 cells/condition from two independent experiments). Statistical significance was determined using a two-way ANOVA followed by Dunnett's multiple comparison test. Error bars represent SEM.

C Tollip siRNA knockdown in SH-SY5Y cells was performed over 72 h, then cells were treated with AO or 25 μM antimycin A (AA) for 2 h, and fixed and immunostained with antibodies specific to VPS35 (green) and TOM20 (red). Colocalisation between VPS35 and TOM20 was not observed. Images were captured at 100× magnification. Zoom insets represent 3.5× magnification.

D HeLa wild type (WT) or VPS35 KO cells transfected with GFP-Tollip were treated with 5 μM antimycin A/10 μM oligomycin (AO) for 2 h, then fixed and immunostained with antibodies specific to GFP (green) and LAMP1 (red).

E HeLa WT or VPS35 KO cells were treated with AO for 2 h and then fixed and immunostained with antibodies specific to Rab7a (green) and LAMP1 (red). Images were captured at 63× magnification.

F Rab7a/LAMP1 colocalisation in HeLa WT and VPS35 KO was assessed by Pearson's correlation. Each data point represents 1 cell, and bar indicates mean value (6–7 cells/condition from two independent experiments). Statistical significance was determined using a two-way ANOVA (Sidak). Error bars represent SEM.

G HeLa VPS35 KO cells stably expressing myc BioID–Tollip (WT) and HA-Parkin were left untreated or treated with AO or AO/100 nM bafilomycin A (BfnA1) for 6 h in the presence of biotin. Cells were lysed and streptavidin pulldowns performed overnight to isolate biotinylated proteins. Proteins in whole-cell extracts (lysate) and pulldowns (SA pulldown) from each condition were then separated by SDS–PAGE and membranes probed with antibodies specific to the indicated proteins or epitope tags.

Data information: Scale bars represent 10 μM.
Source data are available online for this figure.

© 2020 The Authors

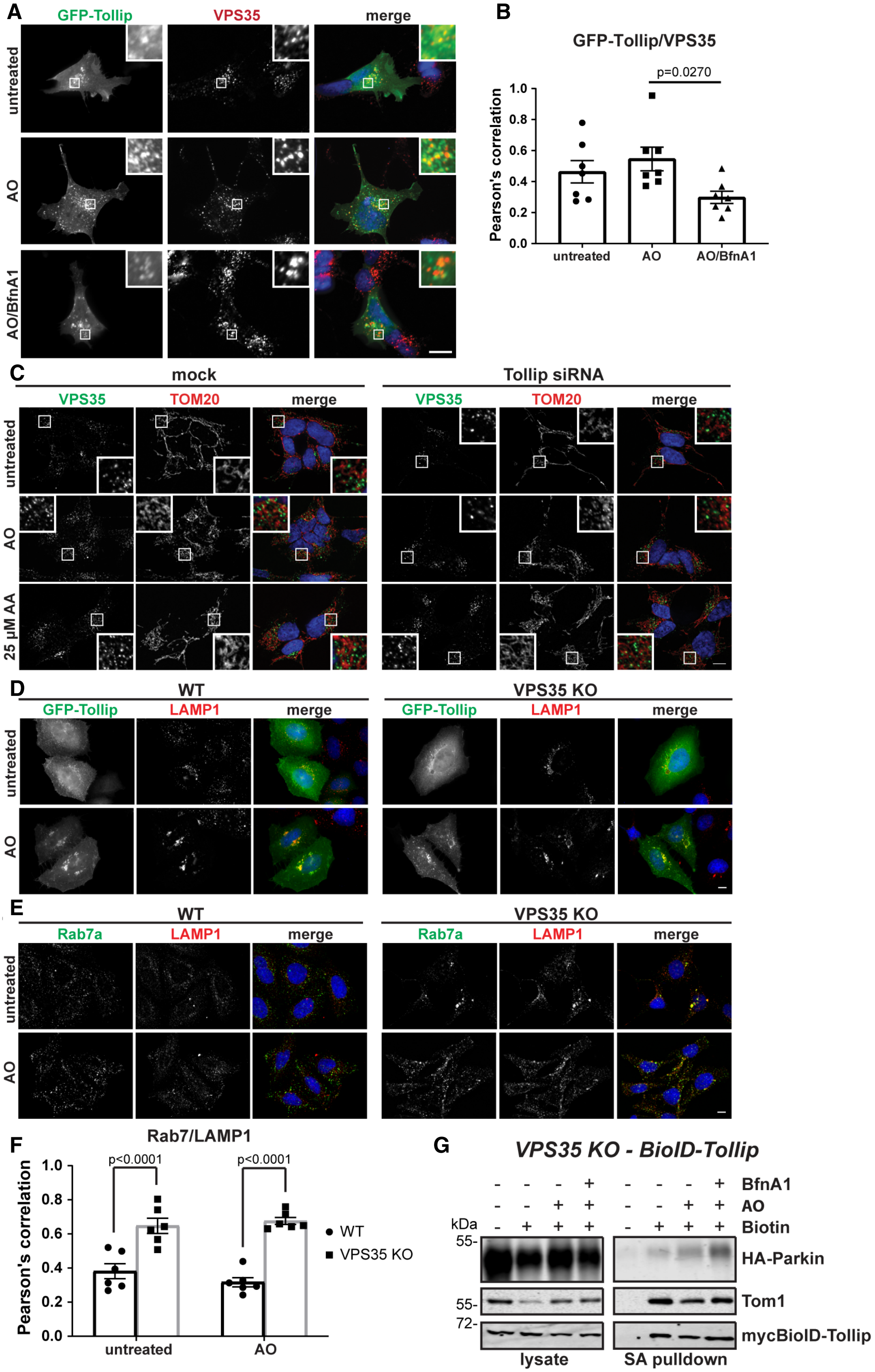

Figure EV5.

© 2020 The Authors
