# Supplementary figures and images for: Tollip coordinates Parkin‐dependent trafficking of mitochondrial‐derived vesicles
Source: EMBO J. 2020 Apr 20;39(11):e102539. doi: 10.15252/embj.2019102539 (PMC7265236; doi:10.15252/embj.2019102539)

**Figure EV1B**

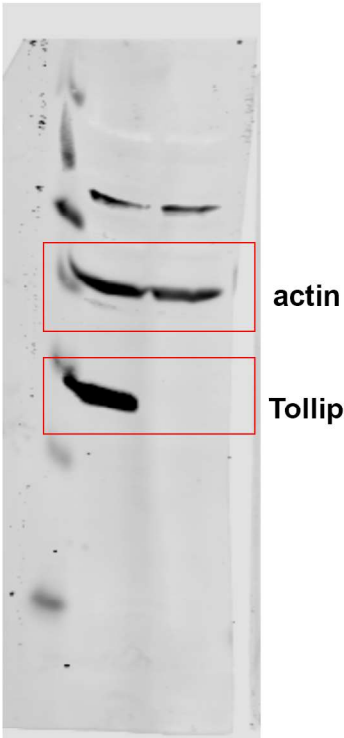

Supplement: Supplementary file 3 — Source Data for Expanded View and Appendix [file EMBJ-39-e102539-s013.zip › embj2019102539-sup-0013-SDataEV/embj2019102539-sup-0013-SDataEVfigs/EMBOJ-2019-102539_SourceDataForFigureEV1B.pdf]

Figure EV3A

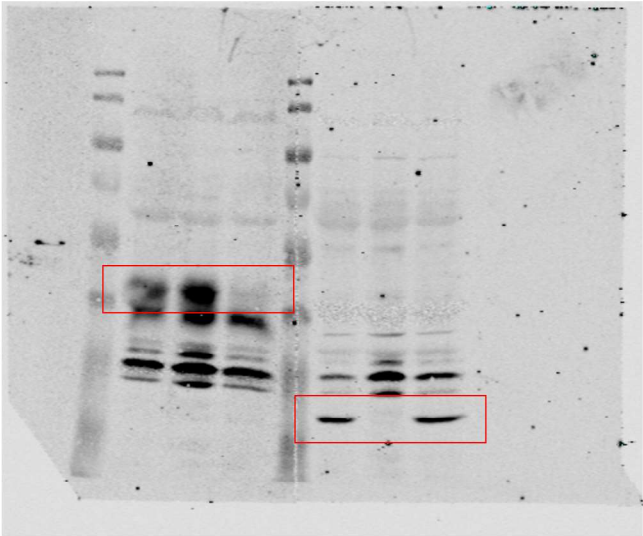

Parkin

Tollip

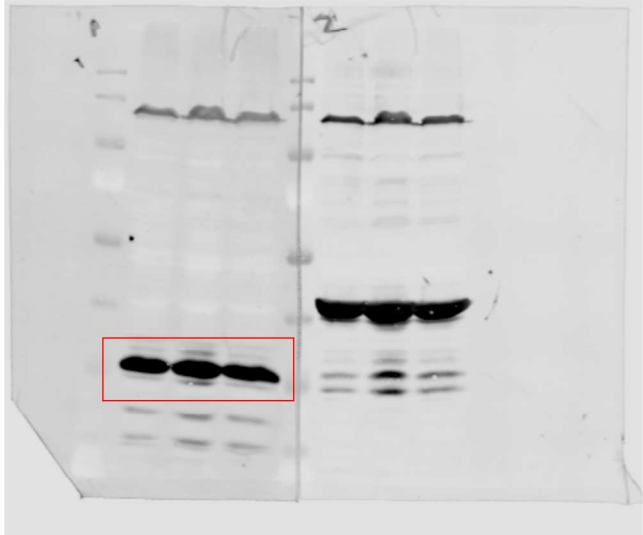

GAPDH

Supplement: Supplementary file 3 — Source Data for Expanded View and Appendix [file EMBJ-39-e102539-s013.zip › embj2019102539-sup-0013-SDataEV/embj2019102539-sup-0013-SDataEVfigs/EMBOJ-2019-102539_SourceDataForFigureEV3A.pdf]

**Figure EV3C**

**Mitofusin-2**

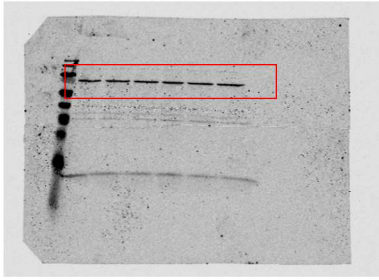

**TOM20**

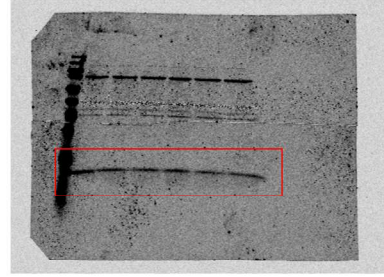

**Parkin**

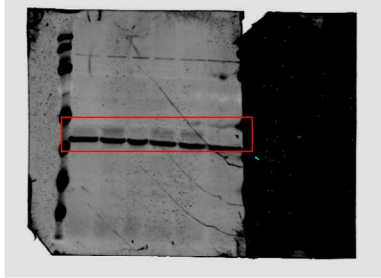

**Tollip**

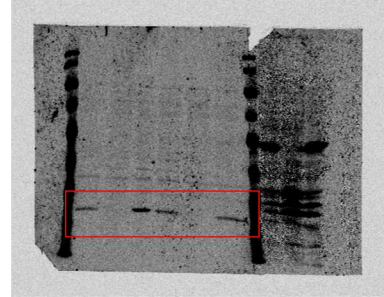

**actin**

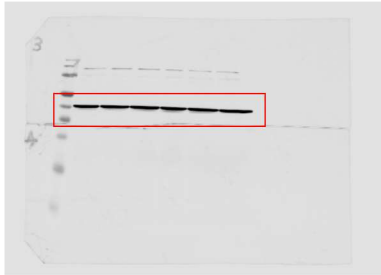

Supplement: Supplementary file 3 — Source Data for Expanded View and Appendix [file EMBJ-39-e102539-s013.zip › embj2019102539-sup-0013-SDataEV/embj2019102539-sup-0013-SDataEVfigs/EMBOJ-2019-102539_SourceDataForFigureEV3C.pdf]

Figure EV4B

SA pulldown

lysate

HA-Parkin

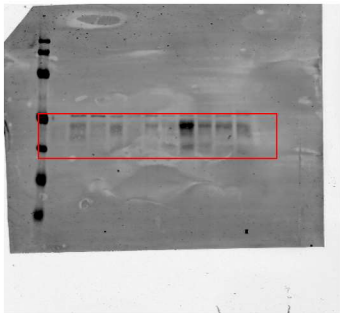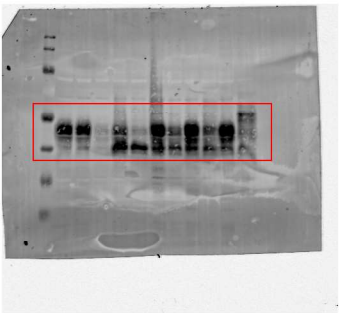

Tom1

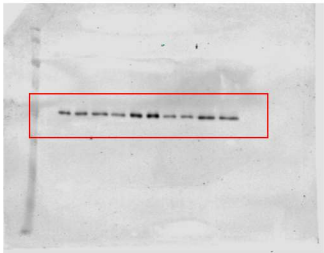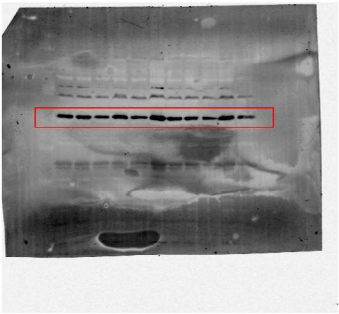

mycBioID-Tollip

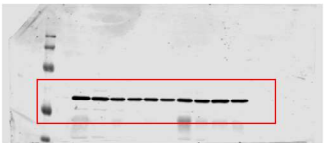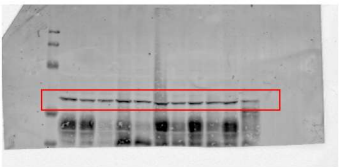

Supplement: Supplementary file 3 — Source Data for Expanded View and Appendix [file EMBJ-39-e102539-s013.zip › embj2019102539-sup-0013-SDataEV/embj2019102539-sup-0013-SDataEVfigs/EMBOJ-2019-102539_SourceDataForFigureEV4B.pdf]

Figure EV5G

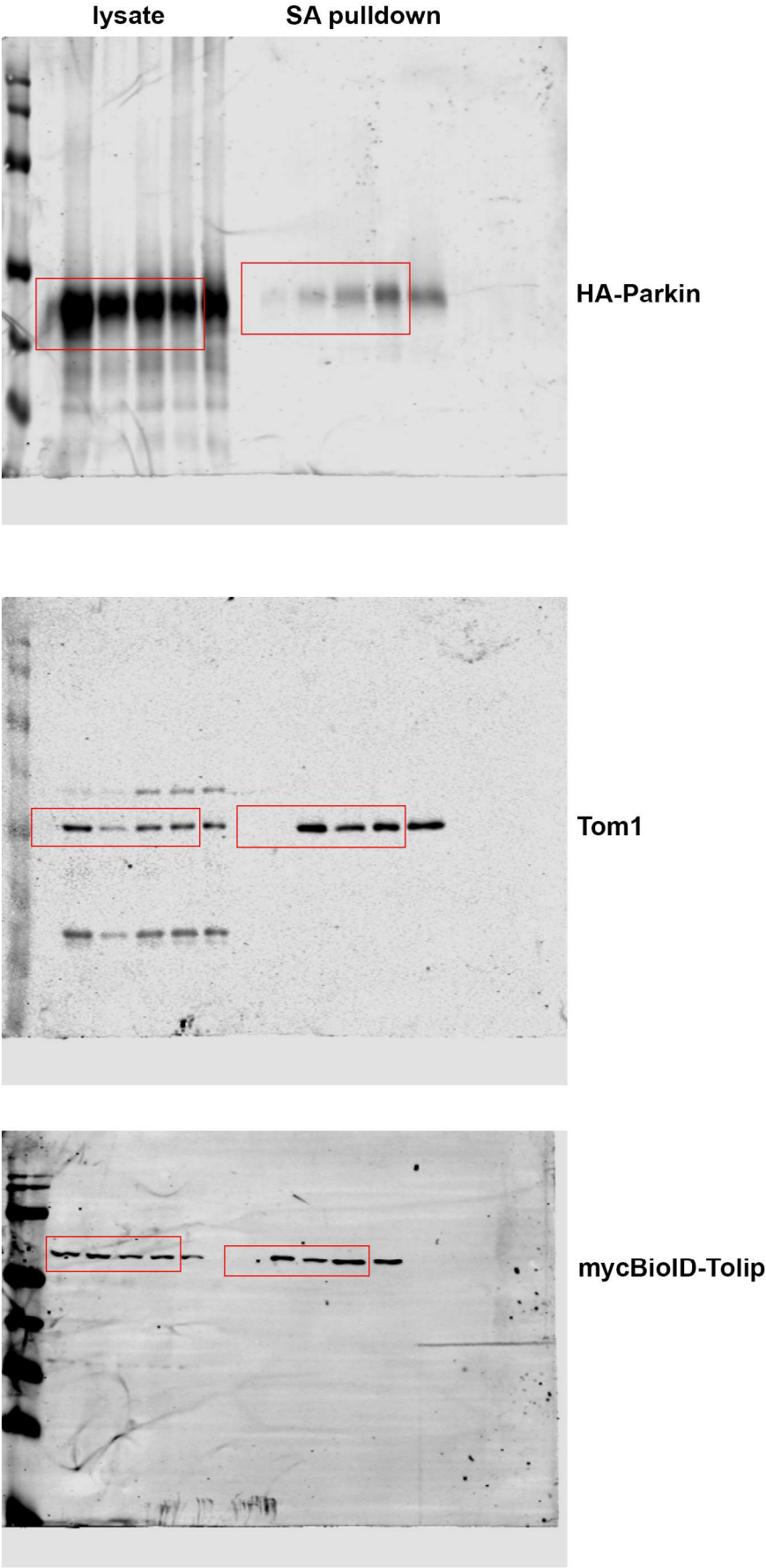

Supplement: Supplementary file 3 — Source Data for Expanded View and Appendix [file EMBJ-39-e102539-s013.zip › embj2019102539-sup-0013-SDataEV/embj2019102539-sup-0013-SDataEVfigs/EMBOJ-2019-102539_SourceDataForFigureEV5G.pdf]

Appendix Figure S1A

K48 ubiquitin

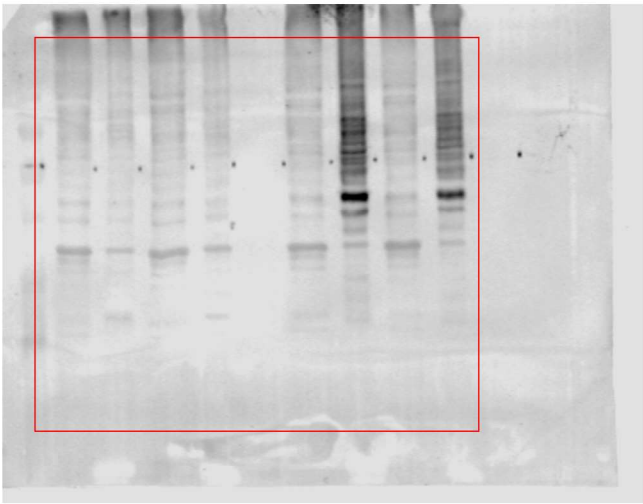

ubiquitin

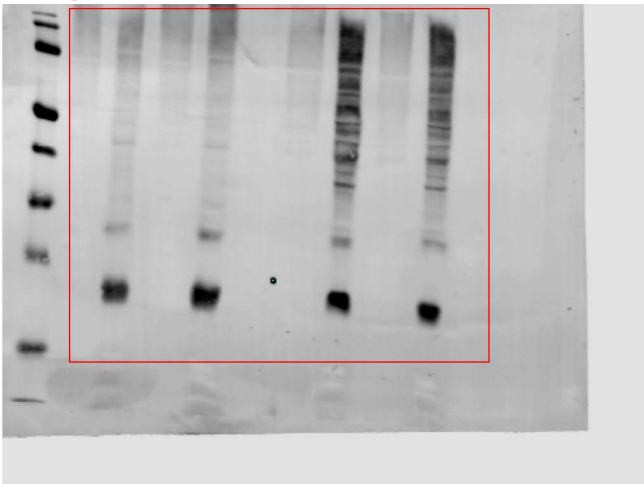

Parkin

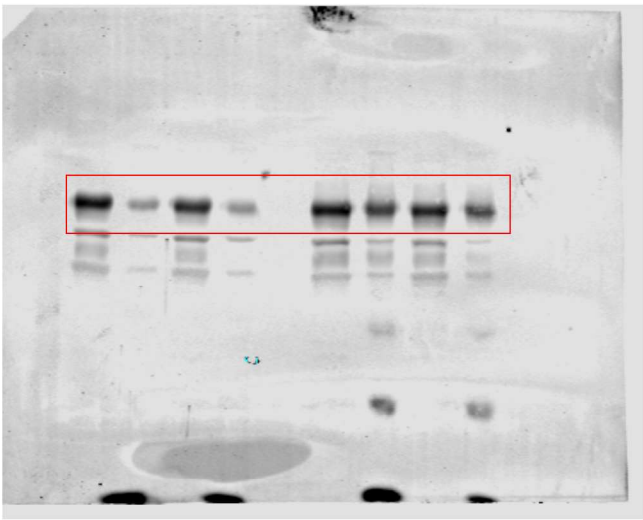

TOM20

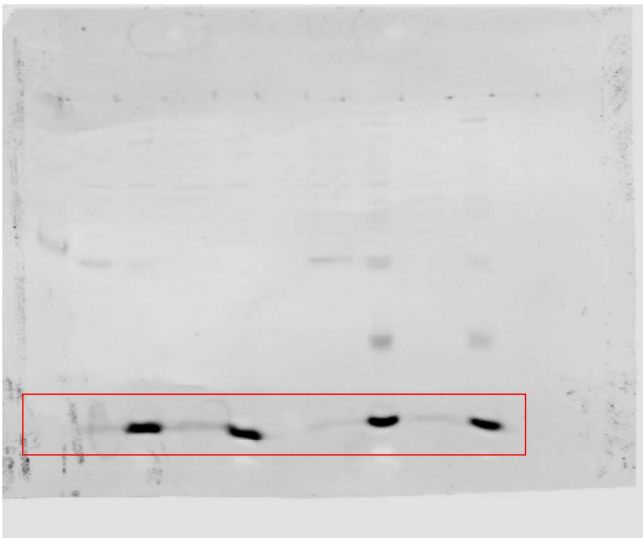

COXII

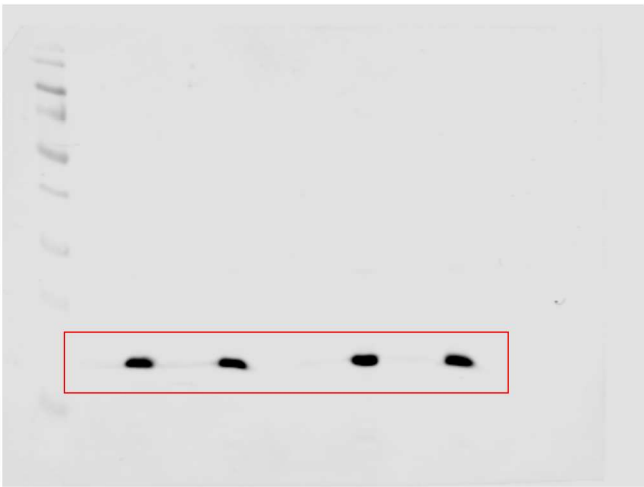

Supplement: Supplementary file 3 — Source Data for Expanded View and Appendix [file EMBJ-39-e102539-s013.zip › embj2019102539-sup-0013-SDataEV/EMBOJ-2019-102539_SourceDataForAppendixFigureS1A.pdf]

Appendix Figure S2D

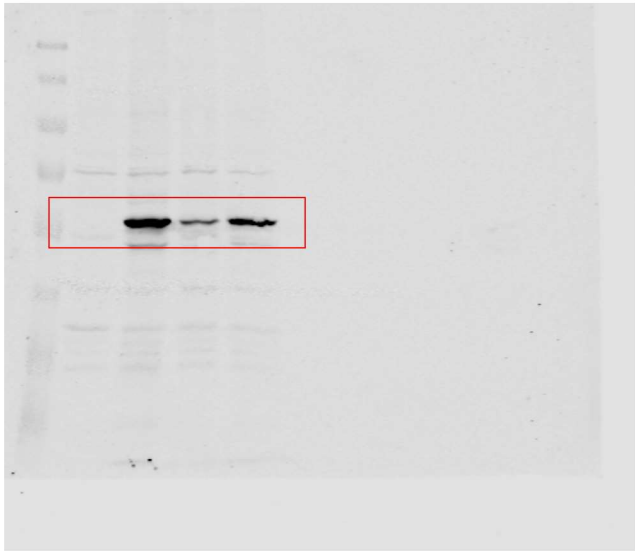

GFP-Tollip

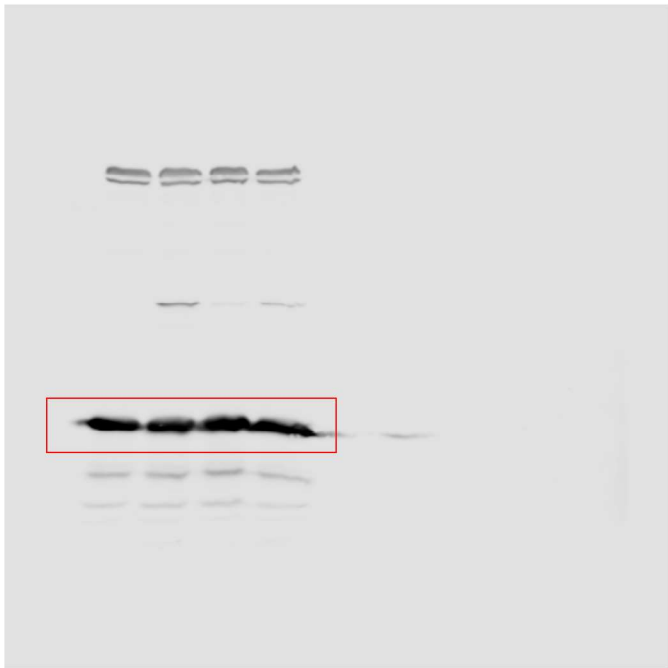

GAPDH

Supplement: Supplementary file 3 — Source Data for Expanded View and Appendix [file EMBJ-39-e102539-s013.zip › embj2019102539-sup-0013-SDataEV/EMBOJ-2019-102539_SourceDataForAppendixFigureS2D.pdf]

Appendix Figure S2E

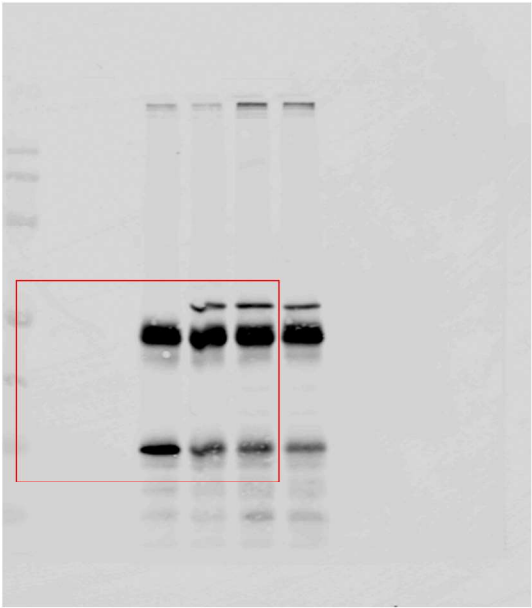

GFP

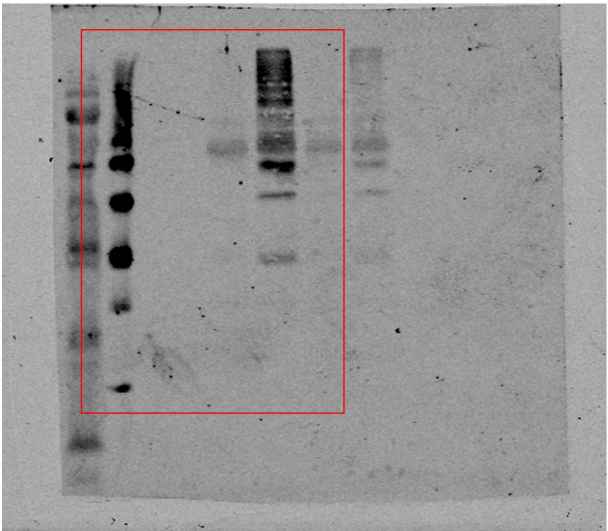

ubiquitin

Supplement: Supplementary file 3 — Source Data for Expanded View and Appendix [file EMBJ-39-e102539-s013.zip › embj2019102539-sup-0013-SDataEV/EMBOJ-2019-102539_SourceDataForAppendixFigureS2E.pdf]

Appendix Figure S5A

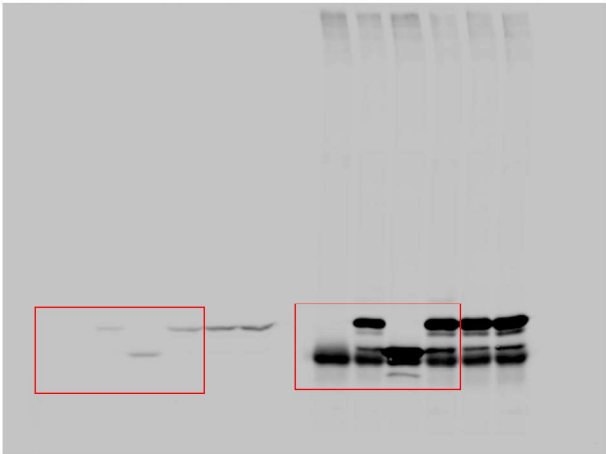

GFP

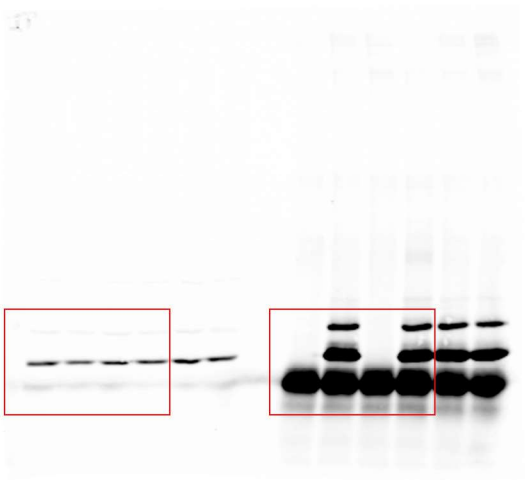

Tom1/Tom1L2

Supplement: Supplementary file 3 — Source Data for Expanded View and Appendix [file EMBJ-39-e102539-s013.zip › embj2019102539-sup-0013-SDataEV/EMBOJ-2019-102539_SourceDataForAppendixFigureS5A.pdf]

Appendix Figure S5B

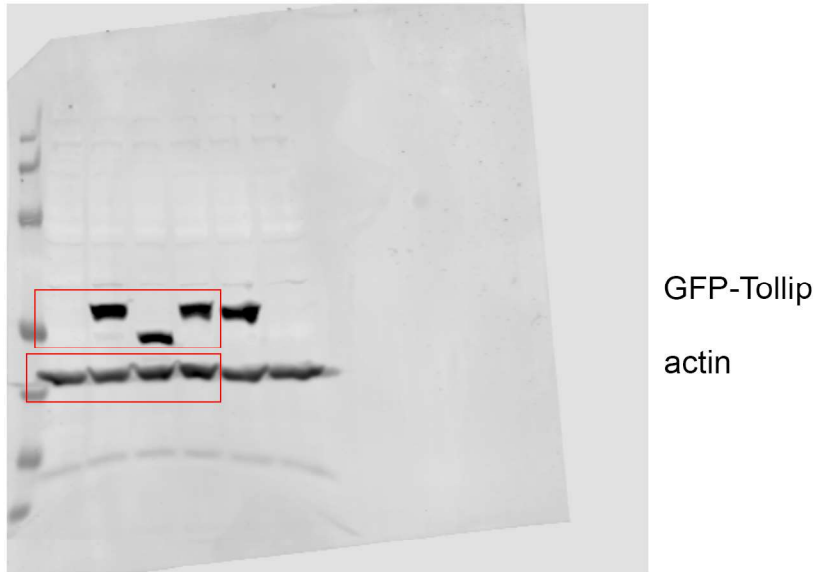

Supplement: Supplementary file 3 — Source Data for Expanded View and Appendix [file EMBJ-39-e102539-s013.zip › embj2019102539-sup-0013-SDataEV/EMBOJ-2019-102539_SourceDataForAppendixFigureS5B.pdf]

Figure 1A

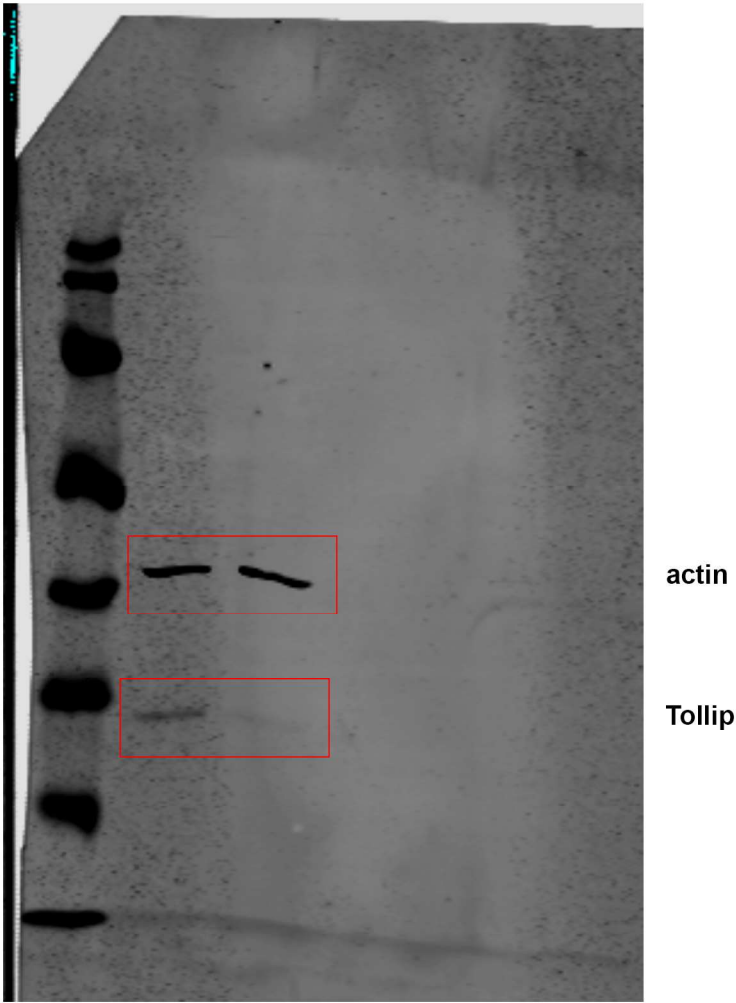

Supplement: Supplementary file 5 — Source Data for Figure 1A [file EMBJ-39-e102539-s003.pdf]

Figure 3A

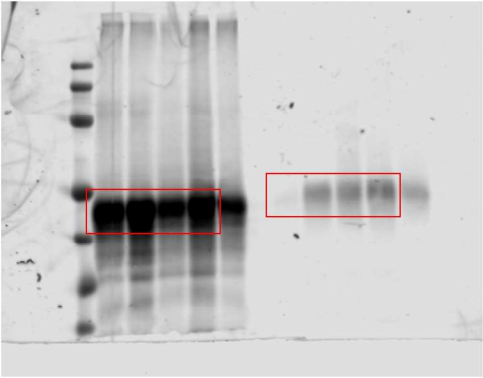

HA-Parkin

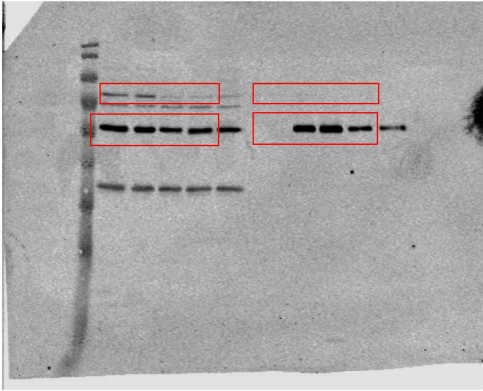

MFN2  
Tom1

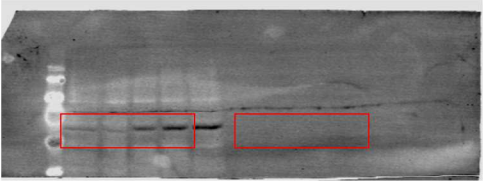

PINK1

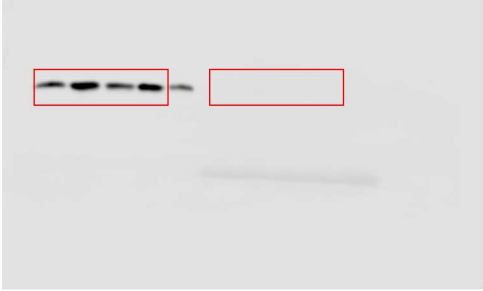

Rab7a

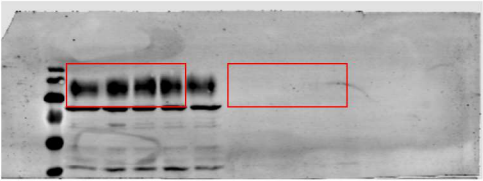

LAMP1

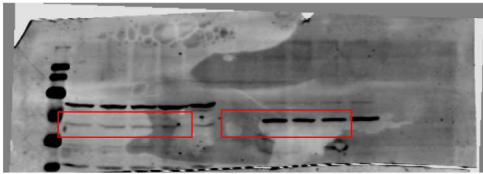

mycBioID-Tollip

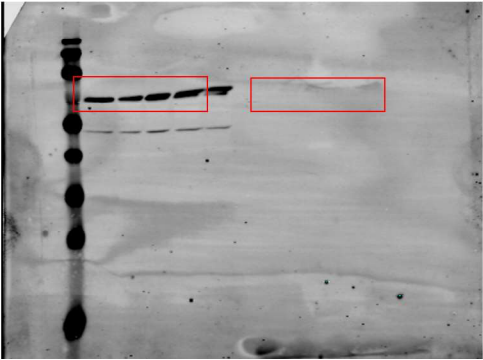

PDH E2/E3bp

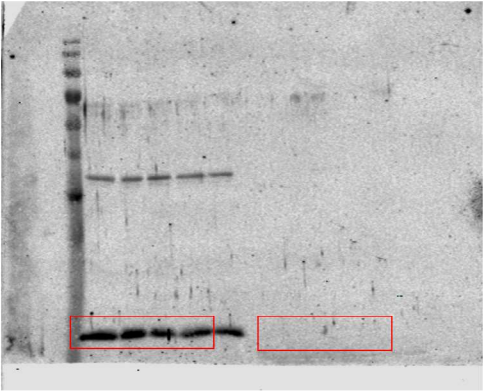

TOM20

Supplement: Supplementary file 6 — Source Data for Figure 3A [file EMBJ-39-e102539-s004.pdf]

Figure 3B

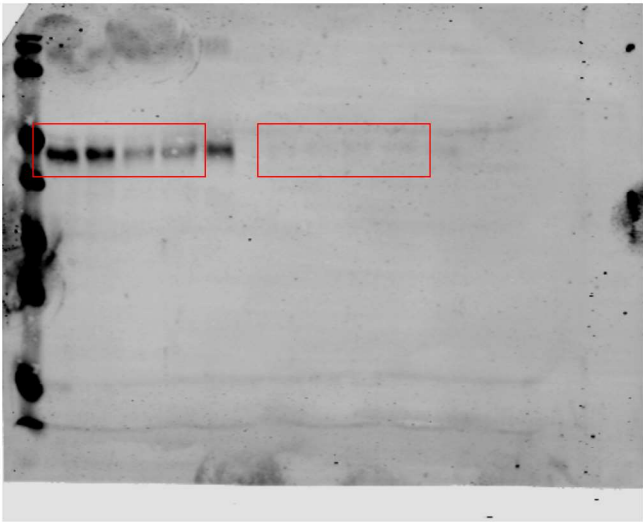

HA-Parkin

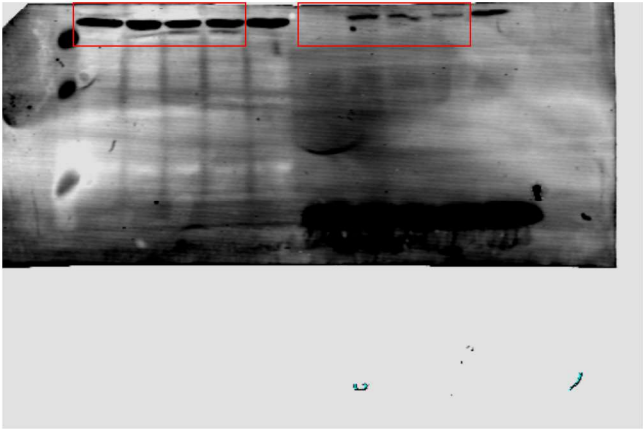

myc-BiolD

Supplement: Supplementary file 7 — Source Data for Figure 3B [file EMBJ-39-e102539-s005.pdf]

**Figure 3C**

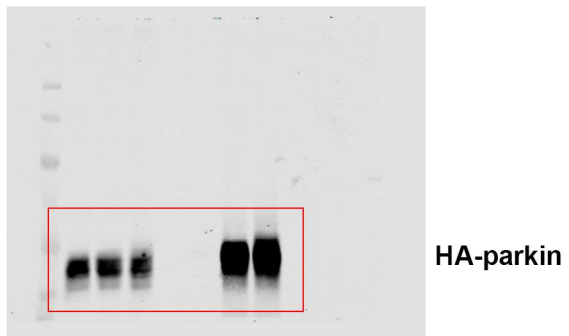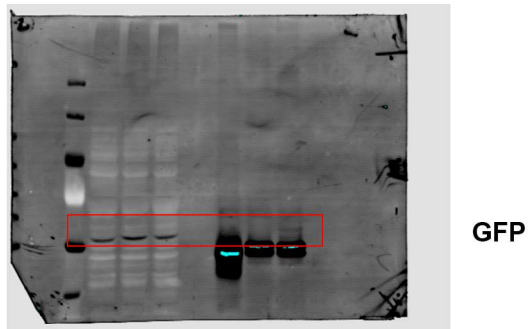

Supplement: Supplementary file 8 — Source Data for Figure 3C [file EMBJ-39-e102539-s006.pdf]

Figure 3D

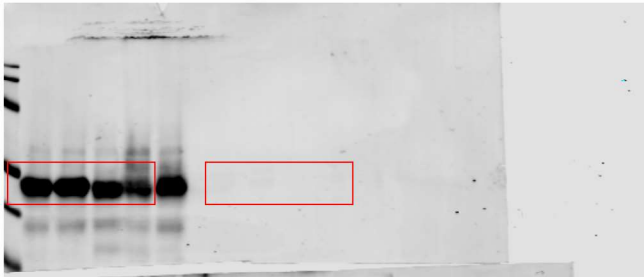

HA-parkin

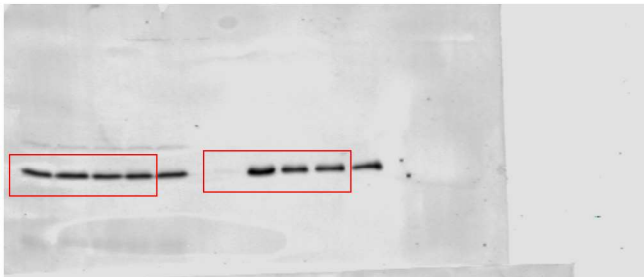

Tom1

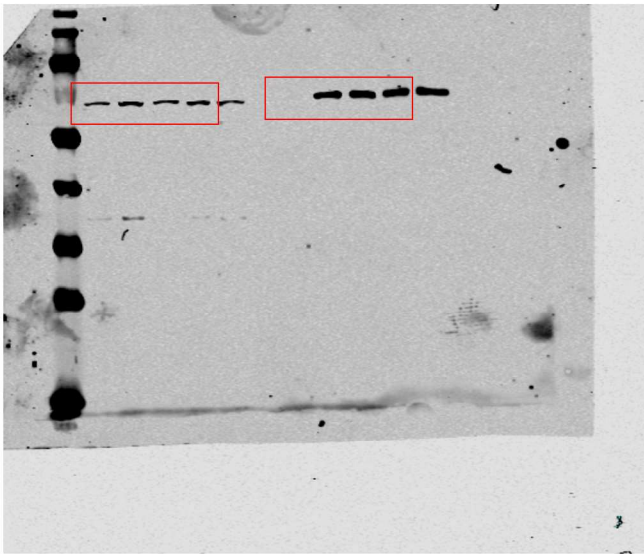

mycBioID-Tollip

Supplement: Supplementary file 9 — Source Data for Figure 3D [file EMBJ-39-e102539-s007.pdf]

Figure 3E

myc-BiolD-Tollip

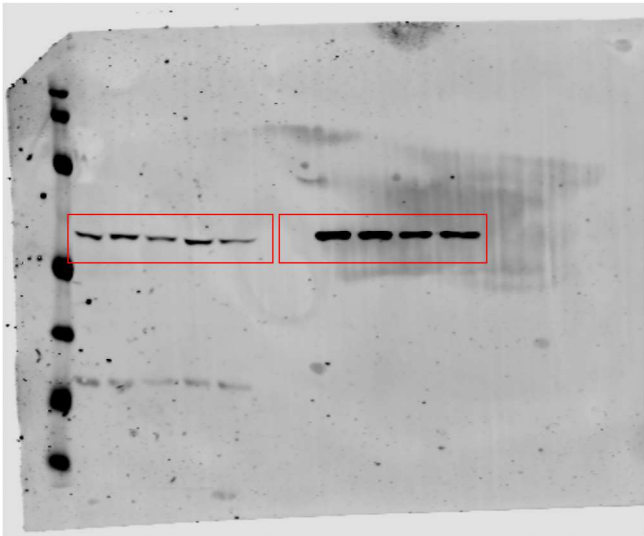

HA-Parkin

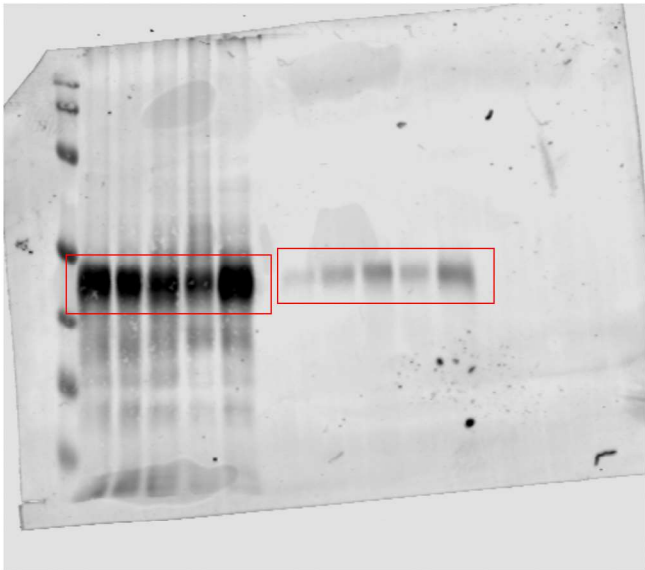

Tom1

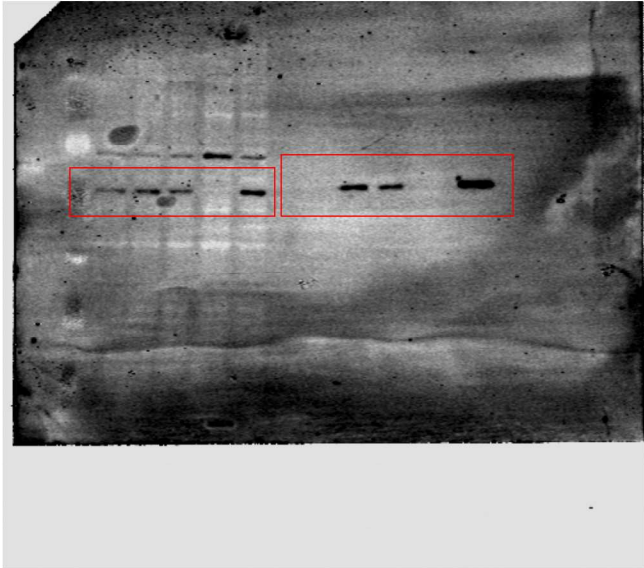

Atg5

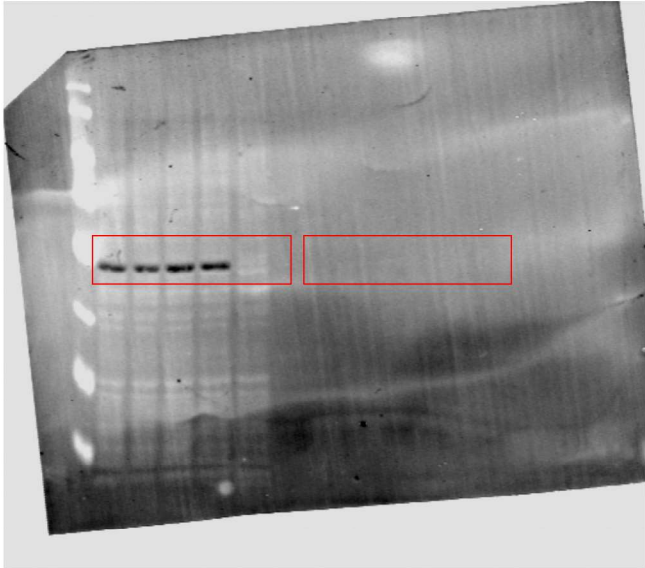

Supplement: Supplementary file 10 — Source Data for Figure 3E [file EMBJ-39-e102539-s008.pdf]

Figure 4B

Tom1

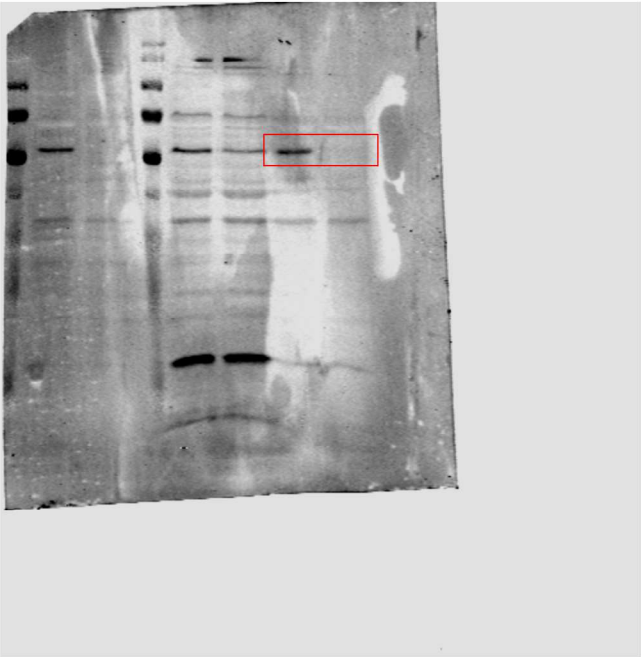

actin

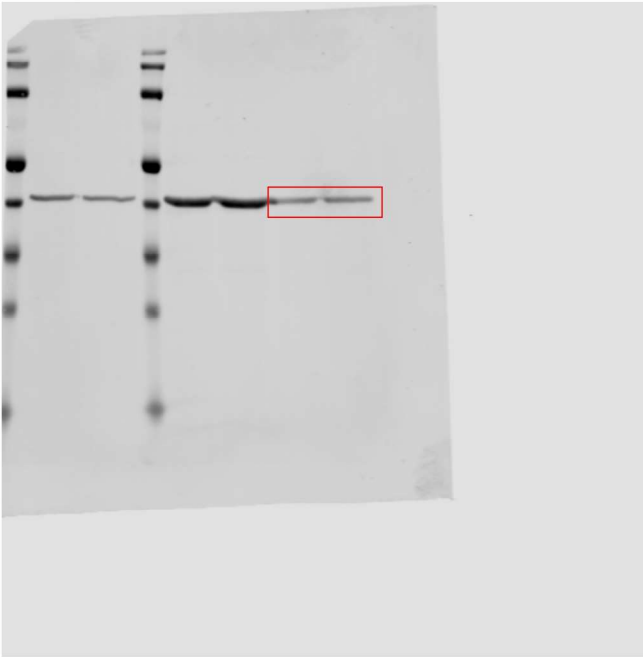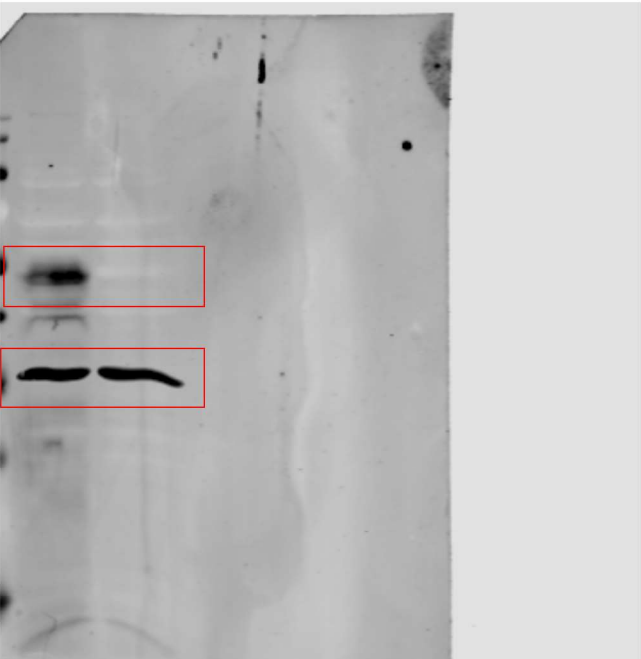

Parkin

actin

Supplement: Supplementary file 11 — Source Data for Figure 4B [file EMBJ-39-e102539-s009.pdf]

Figure 4E

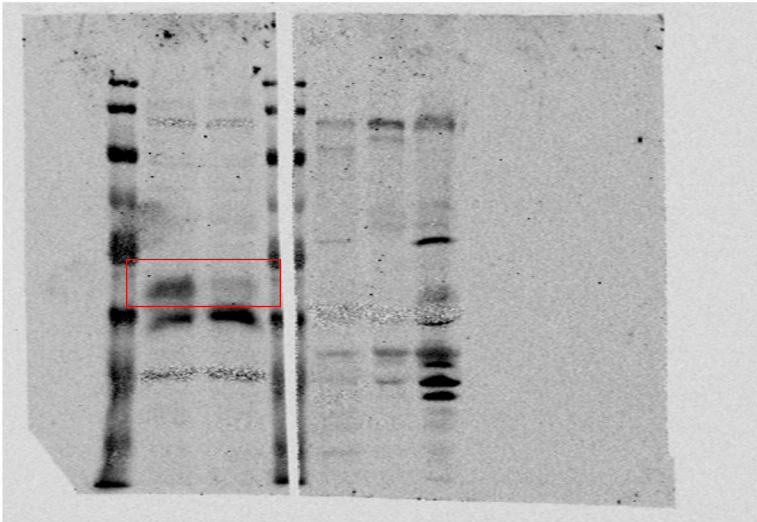

Parkin

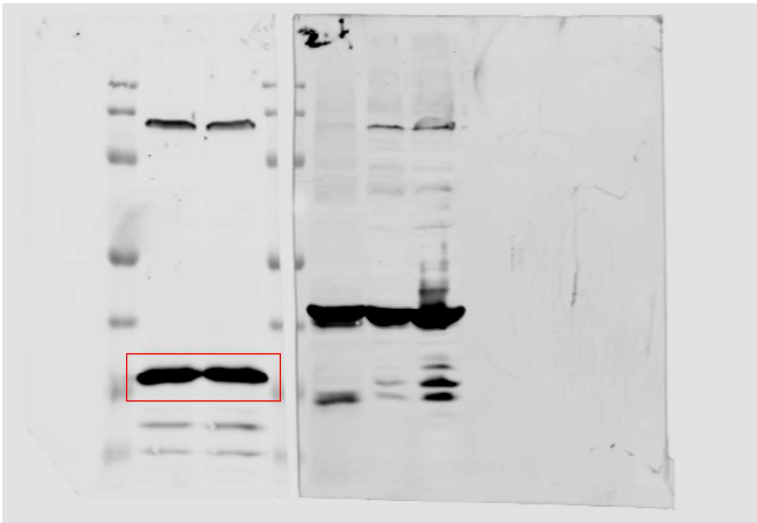

GAPDH

Supplement: Supplementary file 12 — Source Data for Figure 4E [file EMBJ-39-e102539-s010.pdf]

Figure 4H

VPS35

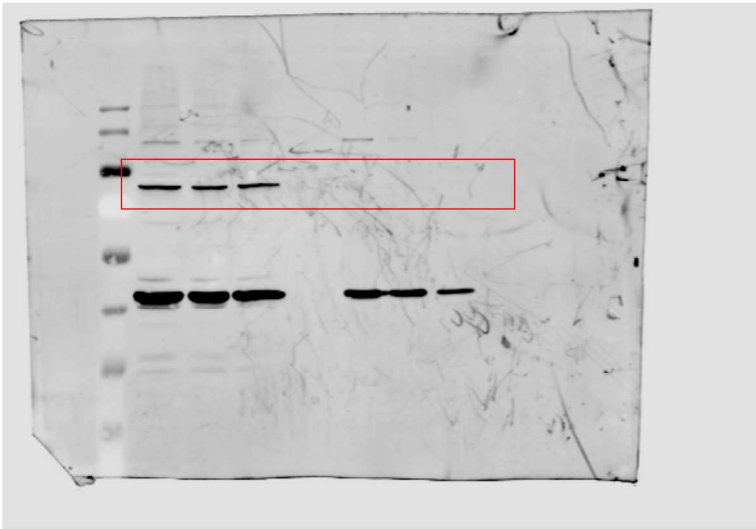

myc

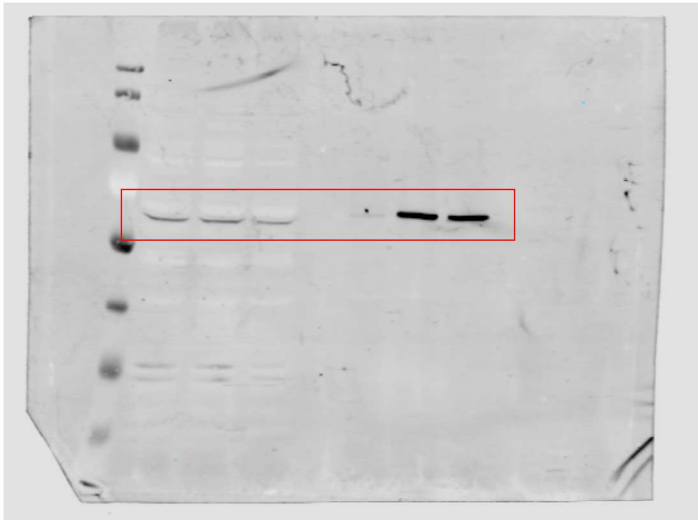

Tom1

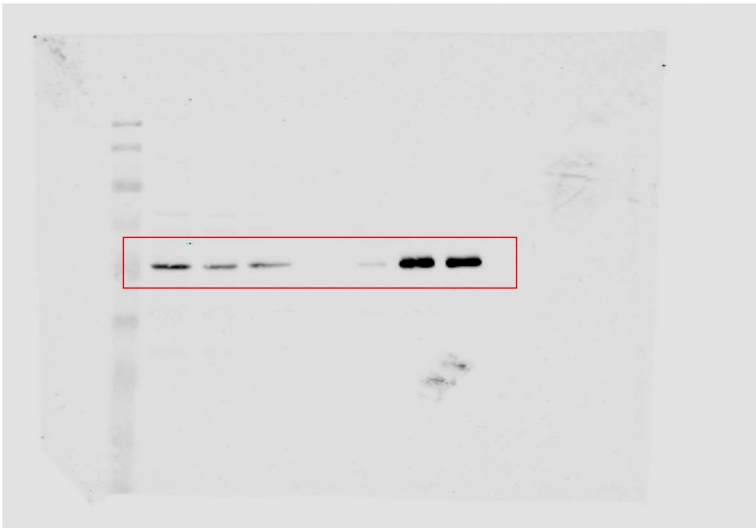

HA

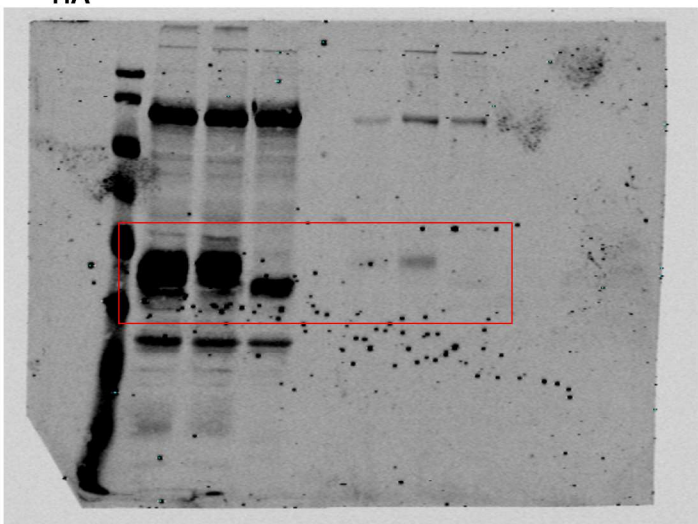

Supplement: Supplementary file 13 — Source Data for Figure 4H [file EMBJ-39-e102539-s011.pdf]

Figure 6G

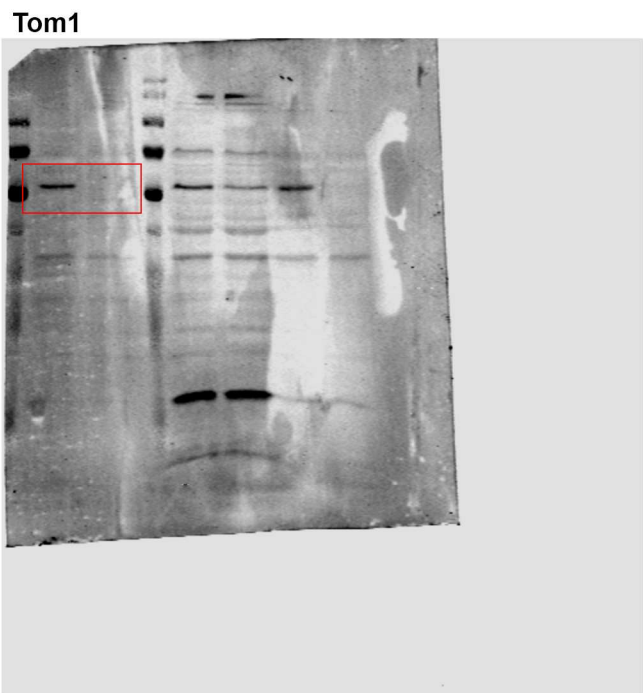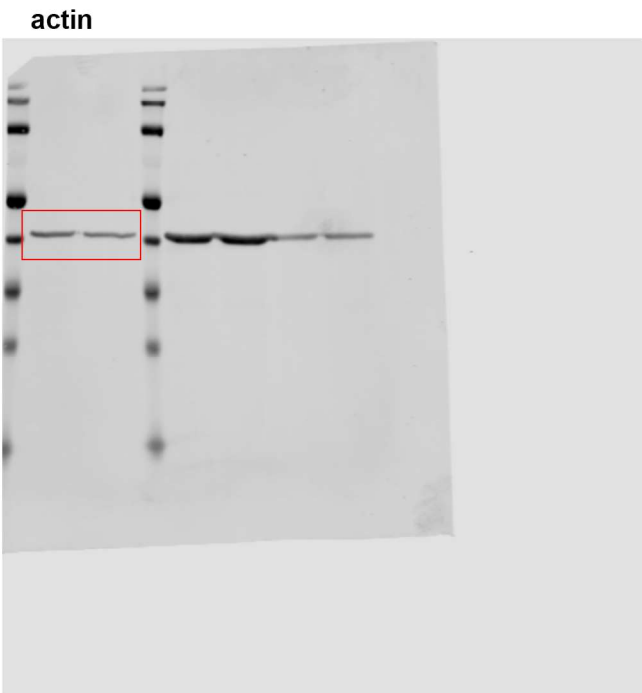

Figure 6I

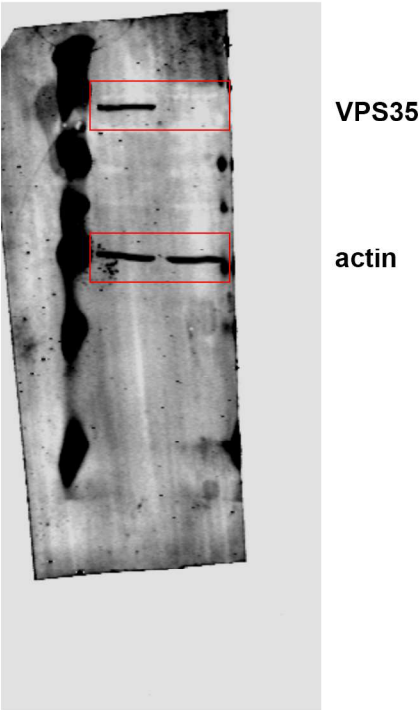

Supplement: Supplementary file 14 — Source Data for Figure 6H,I [file EMBJ-39-e102539-s012.pdf]
